# Supplementary material for: Scalable identification of lineage-specific gene regulatory networks from metacells with NetID
Source: Genome Biol. 2024 Oct 18;25:275. doi: 10.1186/s13059-024-03418-0 (PMC11488259; doi:10.1186/s13059-024-03418-0)
Supplement: Supplementary file 1 — Additional file 1. [file 13059_2024_3418_MOESM1_ESM.pdf]

Supplementary Material

Scalable identification of lineage-specific gene regulatory networks from metacells with NetID

Weixu Wang, Yichen Wang, Ruiqi Lyu and Dominic Grün

Figure S1-S14

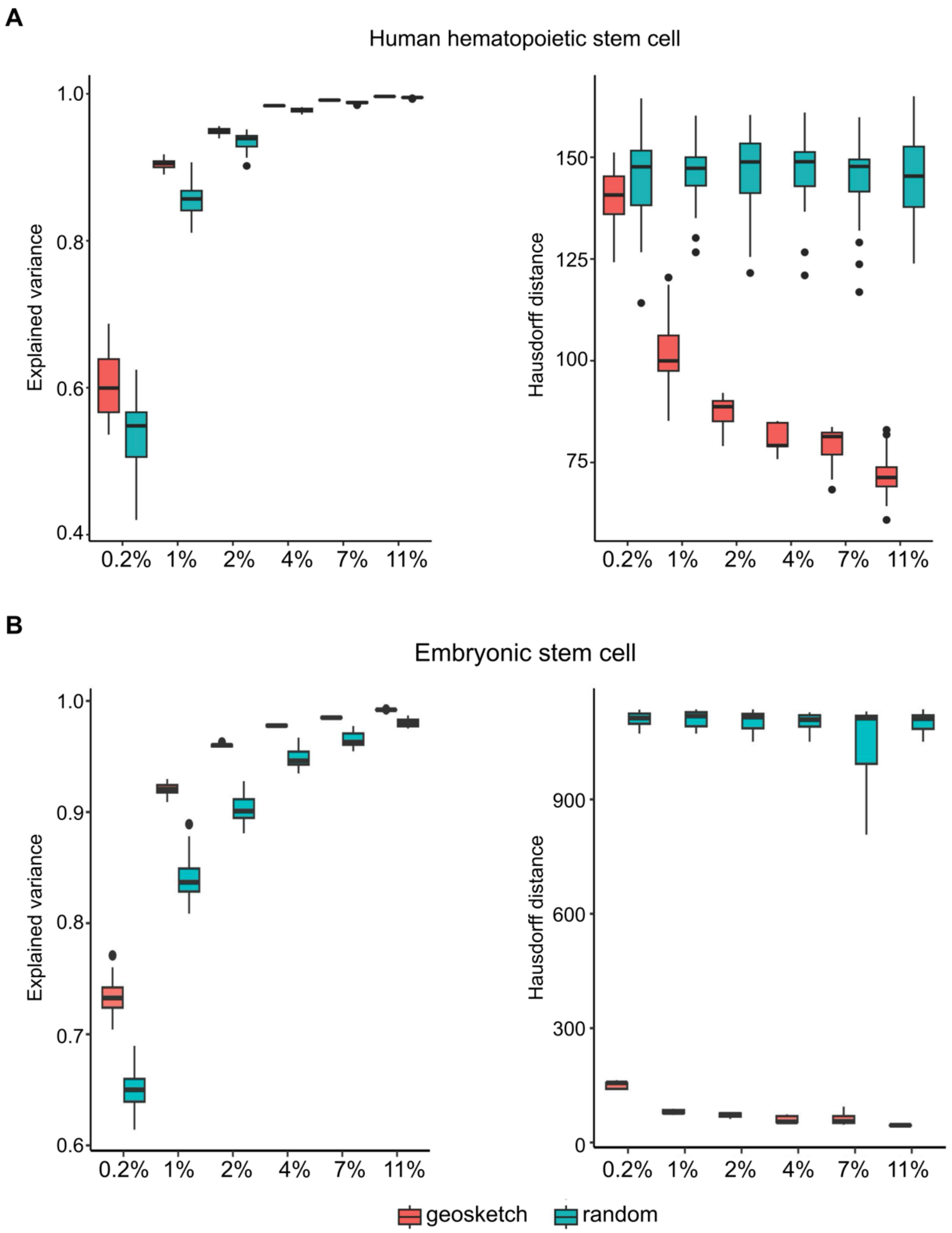

Figure S1. Comparing geosketch sampling and random sampling.

Comparing different sampling strategies for the human adult hematopoietic differentiation [22] and embryonic stem cell [23] dataset.

A Boxplot comparing the Hausdorff distance (left) and Explained Expression Variance (right) of randomly sampled cells (green) and geosketch-sampled cells (red) with 30 repeats for the human hematopoietic stem cell dataset. The x-axis denotes the fraction of sampled cells.

B Boxplot comparing the Hausdorff distance (left) and Explained Expression Variance (right) of randomly sampled cells (green) and geosketch-sampled cells (red) with 30

repeats for the mouse embryonic stem cell dataset. The x-axis denotes the fraction of sampled cells.

In **A** and **B**, the box in the boxplot represents the interquartile range (IQR).The whiskers extend to the smallest and largest values within 1.5 times the IQR. The black line within the box indicates the median.

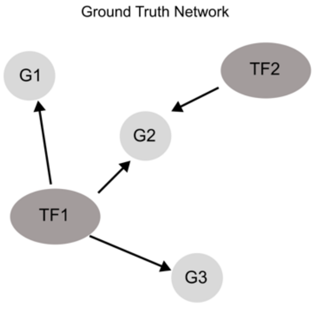

GENIE3 network prediction

Source	Target	True Links	Rank Score
TF1	G1	1	0.9
TF2	G1	0	0.45
TF1	G3	1	0.3
TF2	G2	1	0.1
TF2	G3	0	0.05
TF1	G2	1	0.01

Top K (first four rows)

$$EarlyPrecision = \frac{Hits@K}{K}$$

$$NetworkDensity = \frac{K}{MP}$$

$$EPR = \frac{EarlyPrecision}{NetworkDensity}$$

M : Number of targets
P : Number of regulators
K : Number of edges

Figure S2. Conceptual figure for the early precision rate (EPR).

Early Precision Rate (EPR) is a metric proposed in BEELINE [3] to evaluate early precision compared to a random predictor. EPR is defined as the fraction of true positives among the top-k edges, where k is the number of edges in the ground truth network. In our benchmarking, we consider all predicted top-k edges as positives for all methods, making EPR conceptually similar to precision, which is the fraction of true positive instances among all positive instances. In this figure, we present a toy example with 2 transcription factors (TFs) and 3 targets. In this case, following the described method, EPR is calculated as (3/4) / (4/6) = 1.125.

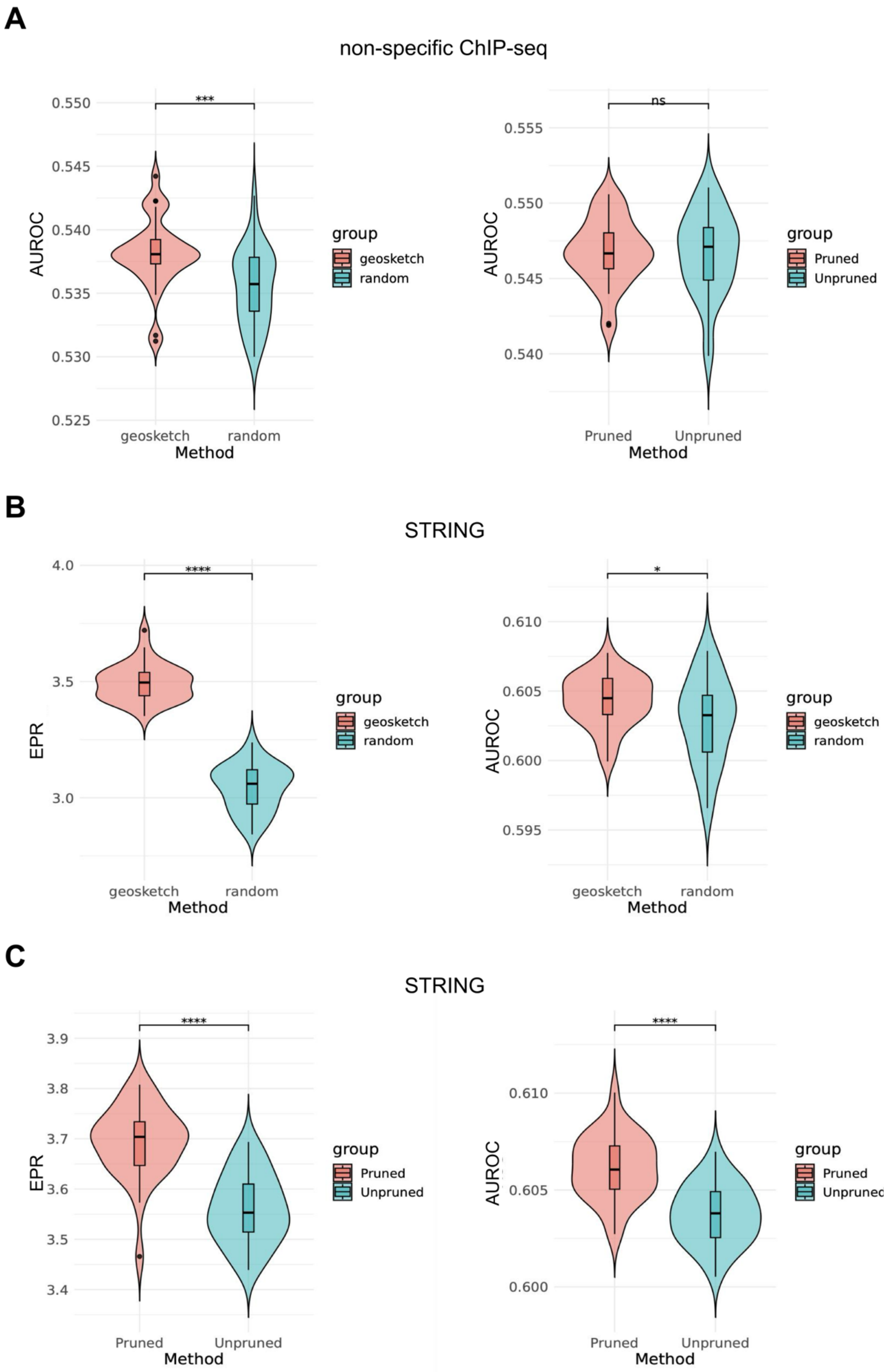
A
non-specific ChIP-seq
AUROC
0.550
0.545
0.540
0.535
0.530
0.525
***
geosketch
random
Method
group
geosketch
random
ns
0.555
0.550
0.545
0.540
Pruned
Unpruned
Method
group
Pruned
Unpruned
B
STRING
EPR
4.0
3.5
3.0
****
geosketch
random
Method
group
geosketch
random
*
AUROC
0.610
0.605
0.600
0.595
geosketch
random
Method
group
geosketch
random
C
STRING
EPR
3.9
3.8
3.7
3.6
3.5
3.4
****
Pruned
Unpruned
Method
group
Pruned
Unpruned
****
AUROC
0.610
0.605
0.600
Pruned
Unpruned
Method
group
Pruned
Unpruned

Figure S3. Comparison of inferred GRN using different sampling strategies and graph pruning for mouse hematopoietic differentiation.

A Violin plot showing the difference in Area Under the Receiver Operating Characteristic Curve (AUROC) when using GENIE3-inferred GRN on geosketch-sampled cells or randomly sampled cells (left) and with pruned KNN graph or unpruned KNN graph (right). Non-specific ChIP-seq data were used as ground truth.

B Violin plot showing the difference in EPR (left) and AUROC (right) when using the GENIE3-inferred GRN on geosketch-sampled cells or randomly sampled cells. STRING data were used as ground truth.

C Violin plot showing the difference in EPR (left) and AUROC (right) when using the GENIE3-inferred GRN on a pruned KNN graph or an unpruned KNN graph. STRING data were used as ground truth.

In **A**-**C**, the box in the violin plot represents the interquartile range (IQR).The whiskers extend to the smallest and largest values within 1.5 times the IQR. The black line within the box indicates the median.

* $P<0.05$, *** $P<0.001$, **** $P<0.0001$, two-sided Wilcoxon rank sum test.

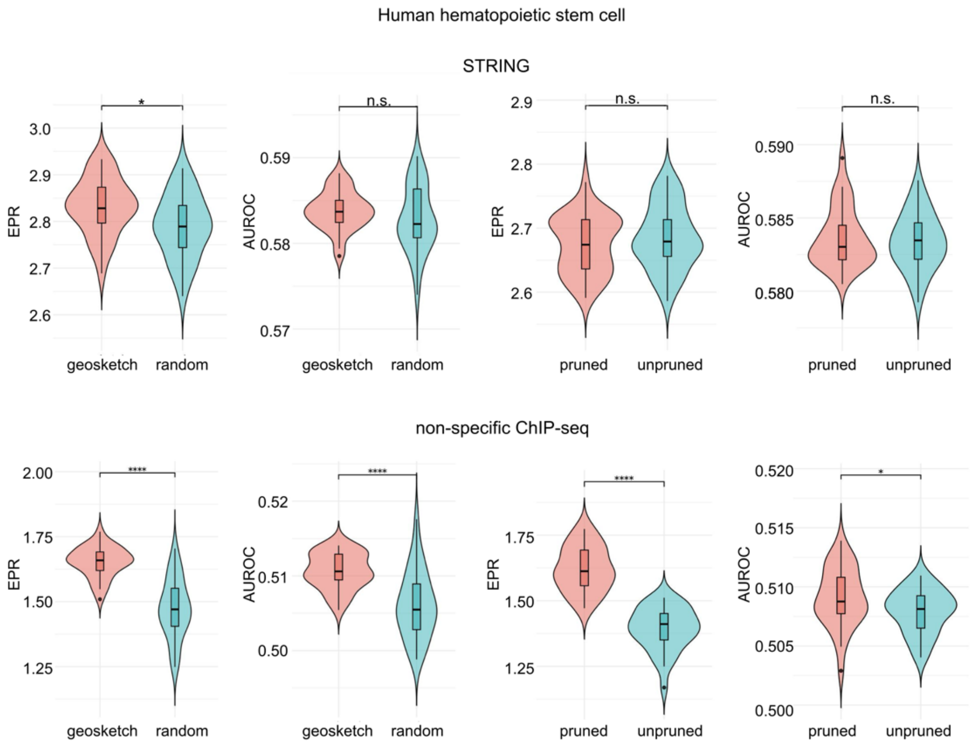

Figure S4. Step-by-step benchmarking of GRN inference to compare the effects of sampling and pruning for human adult hematopoietic differentiation data [2]. We benchmarked sampling (geosketch vs. random) and pruning (pruned vs. unpruned) effects on GRN construction step-by-step. Data are presented as violin plots for both non-specific ChIP-seq data (top) and STRING data (bottom) as the ground truth.

The box in the violin plot represents the interquartile range (IQR).The whiskers extend to the smallest and largest values within 1.5 times the IQR. The black line within the box indicates the median.

* *P*<0.05, **** *P*<0.0001, two-sided Wilcoxon rank sum test.

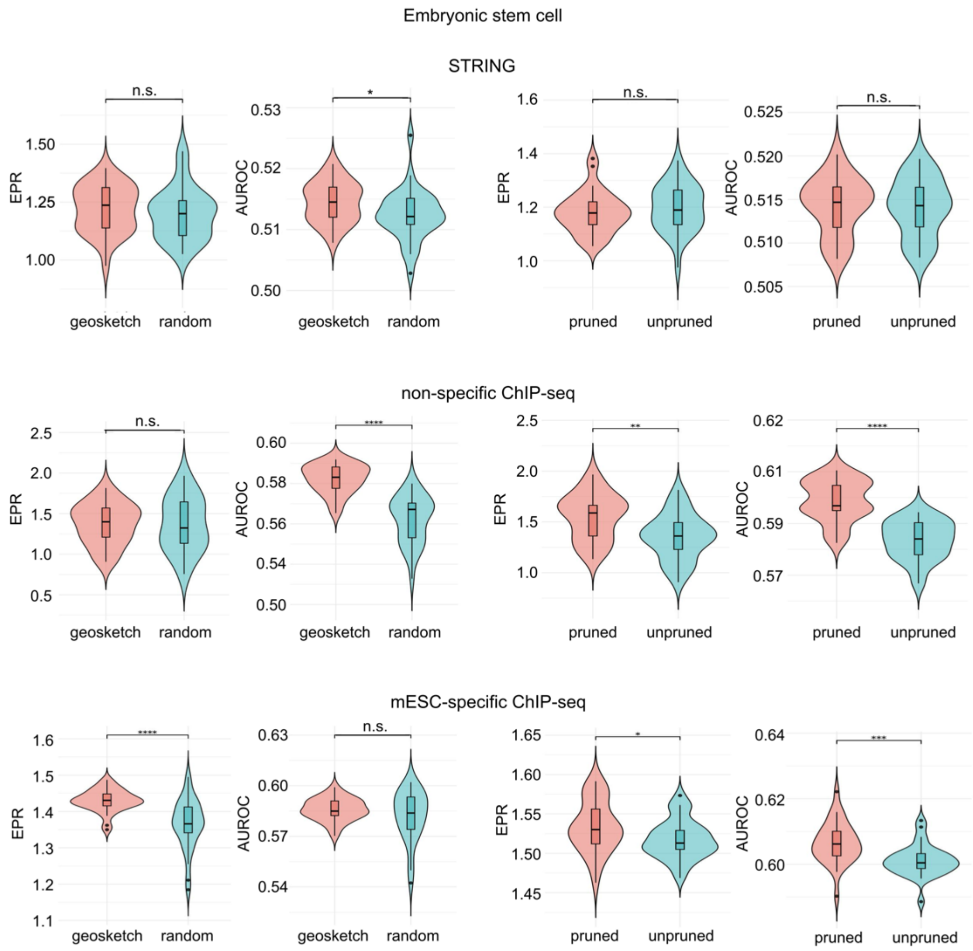

Figure S5. Step-by-step benchmarking of GRN inference to evaluate the effects of sampling and pruning for mouse embryonic stem cell (mESC) data [3].

We benchmarked sampling (geosketch vs. random) and pruning (pruned vs. unpruned) effects on GRN construction step-by-step. Data are presented as violin plots for STRING data (top), non-specific ChIP-seq data (middle) and mESC-specific ChIP-seq data (bottom) as the ground truth.

The box in the violin plot represents the interquartile range (IQR).The whiskers extend to the smallest and largest values within 1.5 times the IQR. The black line within the box indicates the median.

* P<0.05, ** P<0.01, *** P<0.001, **** P<0.0001, two-sided Wilcoxon rank sum test.

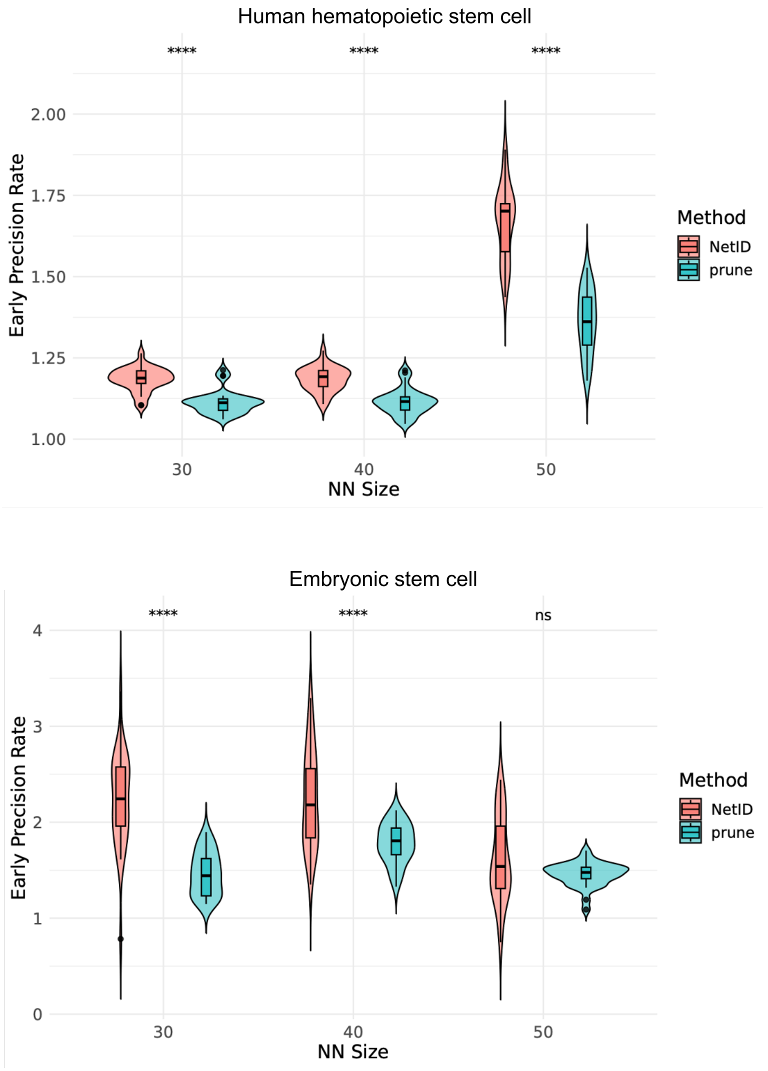

Figure S6. Evaluating the effect of neighbor reassignment for the human adult hematopoietic differentiation and mouse embryonic stem cell (mESC) data.
Violin plots showing the comparison of early precision rate between two different strategies. "NetID" denotes combining geosketch, KNN graph pruning, and neighbor reassignments to build metacell profiles for GRN inference with 30 repeats. "Prune" denotes combining geosketch and KNN graph pruning without neighbor reassignments to build metacell profiles for GRN inference with 30 repeats. The x-axis denotes the number of neighbors. We benchmarked the performance on two biological networks as ground truth: the non-specific ChIP-seq network (human hematopoietic stem cell) and mESC-specific ChIP-seq network (embryonic stem cell).
The box in the violin plot represents the interquartile range (IQR).The whiskers extend to the smallest and largest values within 1.5 times the IQR. The black line within the box indicates the median.
**** P<0.0001, two-sided Wilcoxon rank sum test.

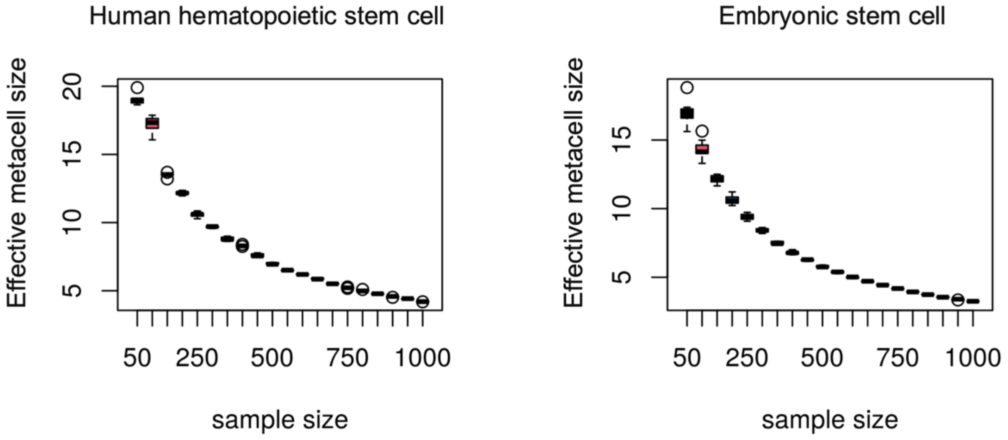

Figure S7. Distribution of the number of partner cells per seed cell for different sample sizes.

Boxplot of the average number of partner cells partner cells per sampled seed cells for human hematopoietic stem cell data (left) and embryonic stem cell data (right). The x-axis denotes the number of sampled seed cells. The y-axis denotes the number of partner cells per seed cell, i.e., the effective metacell size.

The box in the boxplot represents the interquartile range (IQR). The whiskers extend to the smallest and largest values within 1.5 times the IQR. The black line within the box indicates the median.

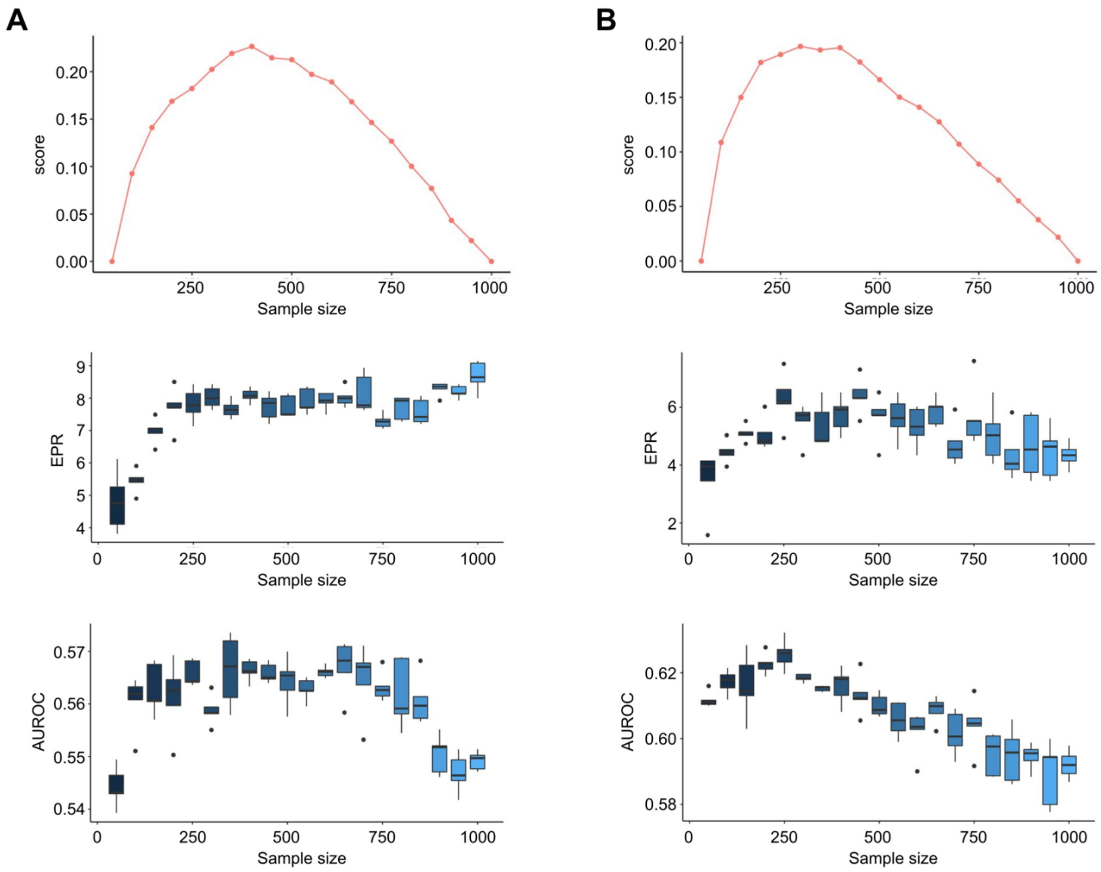

Figure S8. Optimizing the number of sampled seed cells.

A Line plot showing the sample size score as a function of sample size (top). The boxplots show the Early Precision Rate (EPR) (middle) and Area Under the Receiver Operating Characteristic Curve (AUROC) (bottom) for GENIE3-inferred GRNs on different numbers of sampled seed cells. Data are shown for the mouse hematopoietic progenitor dataset and the non-specific ChIP-seq network was used as the ground truth.

B Line plot showing the sample size score as a function of sample size (top). The boxplots show the Early Precision Rate (EPR) (middle) and Area Under the Receiver Operating Characteristic Curve (AUROC) (bottom) for GENIE3-inferred GRNs on different numbers of sampled seed cells. Data are shown for the mouse embryonic stem cell dataset and the non-specific ChIP-seq network was used as the ground truth.

In **A** and **B**, the box in the boxplot represents the interquartile range (IQR). The whiskers extend to the smallest and largest values within 1.5 times the IQR. The black line within the box indicates the median.

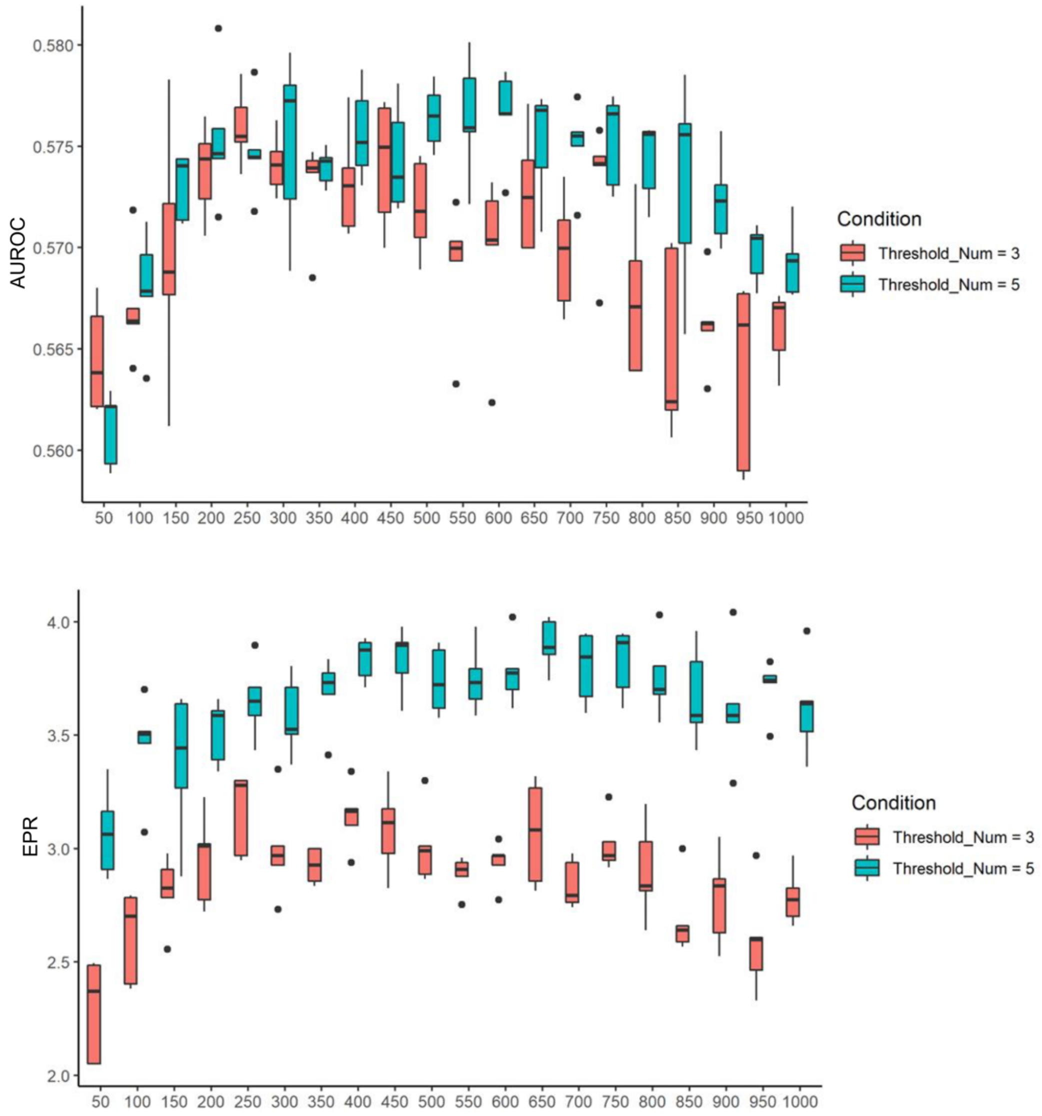

Figure S9. Seed cell filtering effects on GRN inference.
Boxplots showing the difference in Area Under the Receiver Operating Characteristic Curve (AUROC) and Early Precision Rate (EPR) for different thresholds on the number of partner cells to filter seed cells. The x-axis denotes the sampled size, i.e., the number of sampled seed cells. We used the mouse hematopoiesic stem cell gene expression dataset for GRN inference and the non-specific ChIP-seq network as the ground truth.
The box in the boxplot represents the interquartile range (IQR). The whiskers extend to the smallest and largest values within 1.5 times the IQR. The black line within the box indicates the median.

A

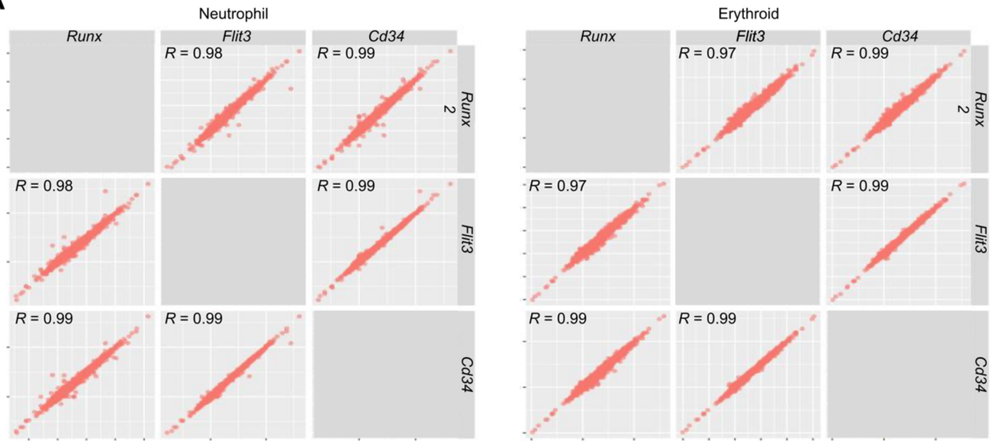

B

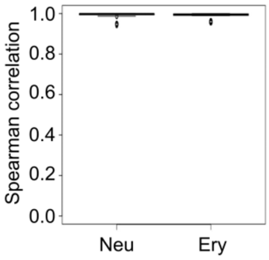

Figure S10. Robustness of GRN inference to root cell selection.

A Pairwise scatter plots display the comparison of lineage-specific gene regulatory networks (GRNs) for the mouse hematopoietic differentiation dataset. We applied NetID for GRN prediction, with cell fate determined by Palantir using different root cell selection methods. Specifically, we tested the stability of inferred GRN weights by selecting root cells based on maximal expression of different marker genes (e.g., *Runx2*, *Flt3*, and *Cd34*) and comparing the results. High correlation between GRN weights across different root cell choices indicates the robustness of the inferred networks to the selection of root cell markers. The terminal states specified are neutrophils and erythroid cells.

B Boxplots display the comparison of lineage-specific gene regulatory networks (GRNs) for the mouse hematopoietic differentiation dataset. We applied NetID for GRN prediction, with cell fate determined by Palantir. Randomly sampled cells from multipotent progenitor cells were used to infer cell fate probabilities. The resulting lineage-specific GRNs were compared, specifying terminal states as neutrophils and erythroid cells.

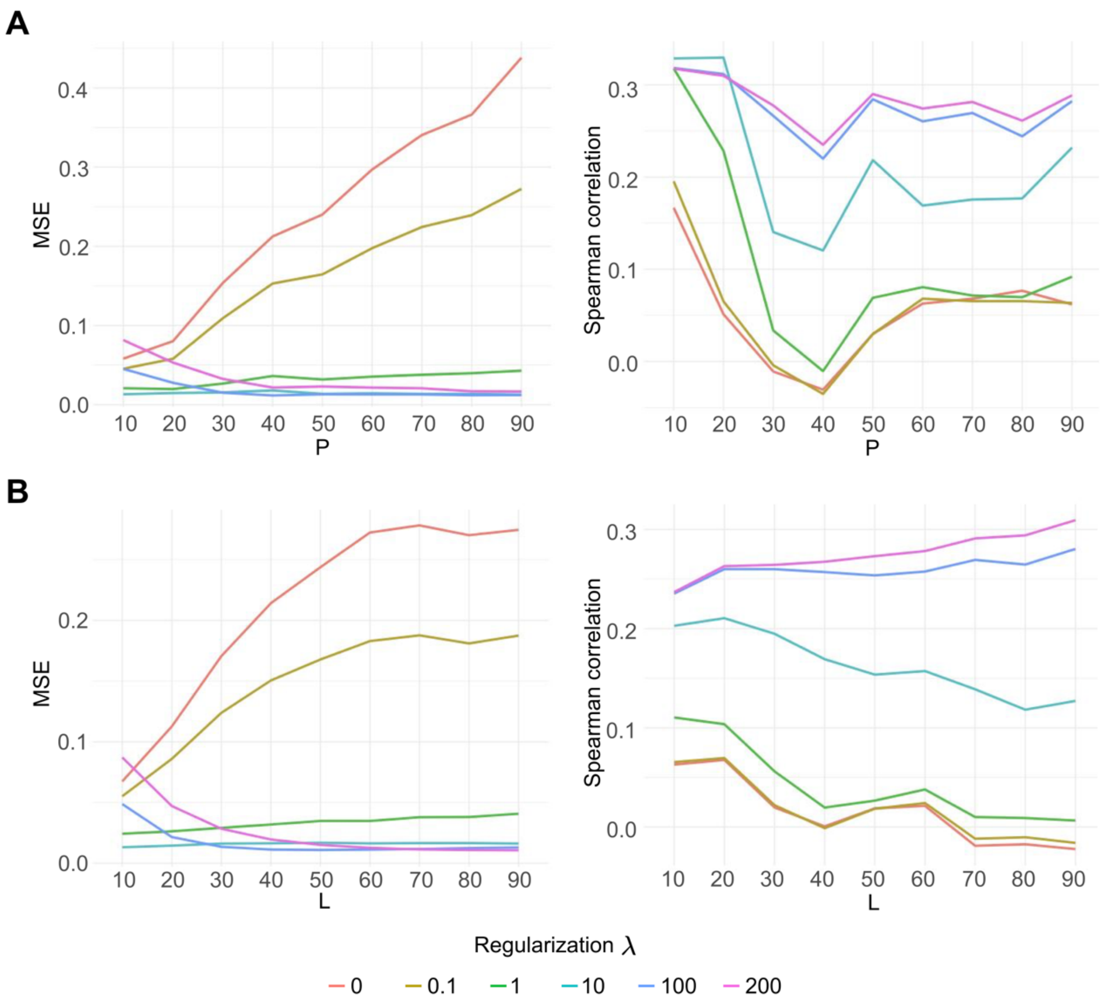

Figure S11. L2 regularization of Granger causal model prevents overfitting.

A Line plots display the predicted mean-squared error (MSE, left) and Spearman correlation (right) on test data (see Results) as a function of the number of regulators. We used six different settings for the L2 regularization parameter.

B Line plots display the predicted mean-squared error (MSE, left) and Spearman correlation (right) on test data (see Results) as a function of the maximum lagged time stamps (*L*). We used six different settings on L2 regularization parameter.

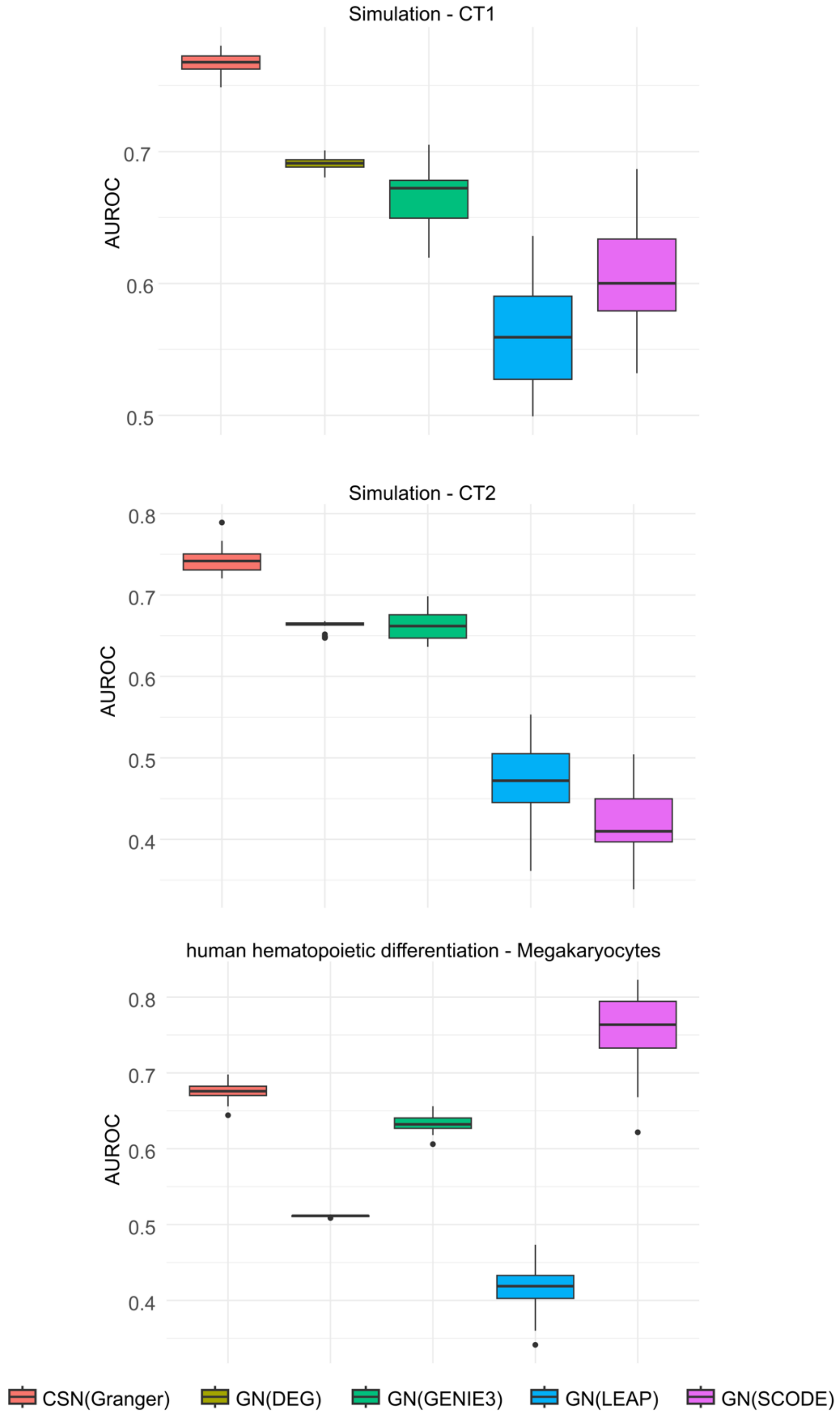

Figure S12. Benchmarking lineage-specific GRN inference.

Boxplots showing the prediction performance evaluated by AUROC for five methods on lineage-specific GRN prediction.

The box in the boxplot represents the interquartile range (IQR).The whiskers extend to the smallest and largest values within 1.5 times the IQR. The black line within the box indicates the median.

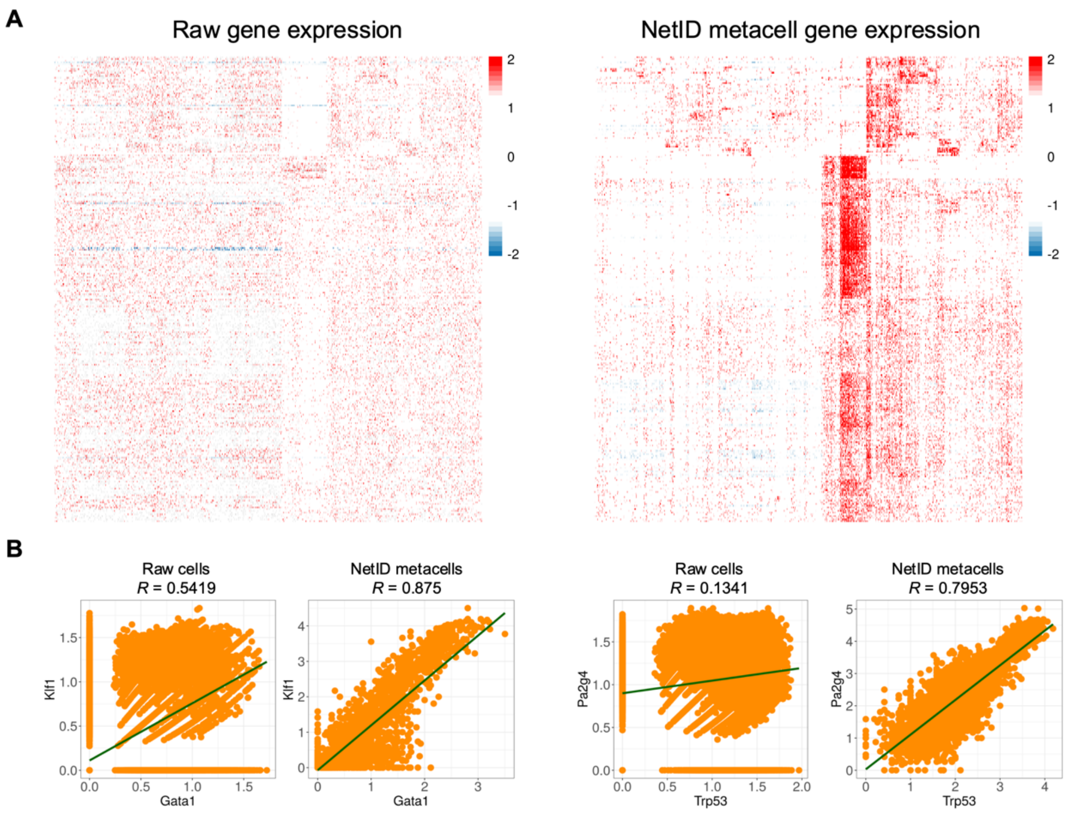

Figure S13. Transcription Factor Expression Analysis for mouse hematopoiesis

A Heatmaps showing raw gene expression (left) and NetID-inferred gene expression for metacells (right) for 271 transcription factors across sampled seed cells for the mouse hematopoiesis dataset [31].

B Scatterplots showing the correlation between *Gata1* and *Klf1* (left) or between *Trp53* and *Pa2g4* (right) using raw gene expression or NetID metacell gene expression.

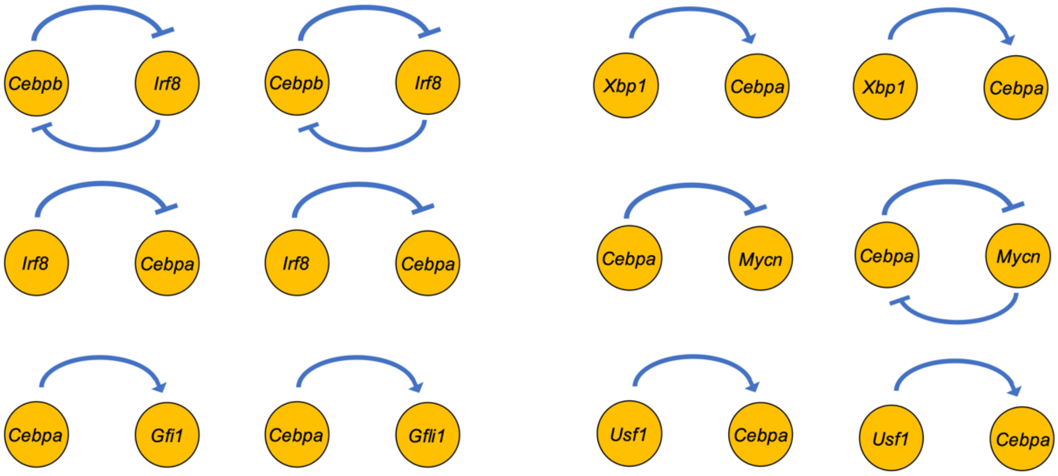

Figure S14. Validation of NetID-predicted gene regulatory relationships.
In addition to the known regulatory links between *Irf8* and *Cebpa*/*Cebpb* described in the results section, NetID correctly predicted positive regulatory relationships from *Cebpa* to *Gfi1* [52], *Usf1* to *Cebpa* [53], *Xbp1* to *Cebpa* [55], and the negative regulatory relationship from *Cebpa* to *Mycn* [54].
