# Supplementary material for: Scalable identification of lineage-specific gene regulatory networks from metacells with NetID
Source: Genome Biol. 2024 Oct 18;25:275. doi: 10.1186/s13059-024-03418-0 (PMC11488259; doi:10.1186/s13059-024-03418-0)
Supplement: Supplementary file 3 — Additional file 3. [file 13059_2024_3418_MOESM3_ESM.pdf]

Review history

First round of review

Reviewer 1

The paper "Scalable identification of lineage-specific gene regulatory networks from metacells with NetID" by Wang and Gruen presents a novel regulatory network inference algorithm for scRNA-Seq data, called NetID. The algorithm is in effect, an ingenious "stitch-up" of many different methods. One key element is the construction of metacells ,(Baran Y et al Genome Bio 2019) using seeds selected with the "geosketch" algorithm from Berger's lab, although oddly enough this contradicts what is stated in Methods, where the authors imply that a 2nd method implemented in Seurat called "SeuratSketching" is used. The use of meta-cells makes sense as the authors aim to optimize the trade-off between cell-numbers and the sparsity/noise of the profiles used for inference. Once the meta-cells are inferred, the author rely effectively on the published GENIE3 method to infer a global regulatory inference network, which the authors then use as a "scaffold or prior network" to be integrated with lineage-specific networks that are inferred using Granger causality ridge regression. In all honesty though, this Granger-causality step is probably only a minor refinement, because unlike what the authors are trying to imply, there is substantial lineage-specific information in the GENIE3 derived network, as long as GENIE3 is applied sensibly over cell populations where the underlying variation is associated with bifurcating cell-differentiation processes. The authors use both simulated as well as real datasets to assess performance, but the benchmarking is mainly against methods that perform imputation on scRNA-seq data, and not to other existing regulatory network inference methods (see further below). The authors use a number of evaluation frameworks and "ground-truths" but other potentially better alternatives are ignored.
Overall, while I think that NetID should be of interest to the single-cell community, its value seems mostly confined to the construction of metacells and the GENIE3 network, both of which are based on existing algorithms, which would hence seem to justify more of a software or "applications note" paper. Indeed, the "improvement" over existing methods as shown in this MS is only fairly marginal, as there are cases where some of the imputation methods perform better (e.g. Fig4B msHSC-specific ChIP-Seq and non-specific ChIP-Seq), and some benchmarks are missing. I am also concerned about comparing methods based on only 10 repeated runs. Surely, one should consider more runs to make sure that non-significant differences are truly non-significant. The Granger-causality step method also does not necessarily add much novel biological insight as the TFs that are being recovered are well-known and could have been found by much simpler or other existing methods (e.g. integrating DEG-analysis or GENIE3 with a PPI-network). Another concern is the lack of clarity in the implementation of methods, as exemplified by the example above when selecting seed cells to build metacells.
Below are my major concerns, in the hope these can be useful:
Major concerns:
1) Is the Granger-causality step really needed?: Throughout the MS, the authors seem to imply that the global network inferred with GENIE3 contains no information about lineage-specific regulatory networks, which is why the authors subsequently apply this Granger-causality ridge regression step to seemingly infer lineage-specific regulatory networks. However, I can't possibly agree with this statement. If you apply GENIE3 to a scRNA-Seq data encompassing say a multipotent progenitor population as well as differentiated cells for all downstream lineages, most of the variation in such a dataset would obviously capture differences between the lineages, and that information gets encoded in the global regulatory network. To then identify lineage specific subnetworks would only be a matter of studying the gene-expression patterns for cells mapping to a given lineage in the context of this global network, which would reveal lineage-specific subnetworks. Hence, the rationale for Granger-causality ridge regression is not entirely clear to me: I understand that you can use it to infer potential regulatory relations within a lineage,

relying on the gradients of expression within a lineage (ordered by pseudotime). However, this would only constitute a refinement. In summary, I feel that the Granger-causality step needs to be benchmarked against an alternative approach which studies the expression patterns of lineage-specific cells on the inferred GENIE3 global network.
2) Evaluation frameworks lack clarity: the authors sometimes use specific ChIP-seq data, sometimes non-specific ChIP-Seq data and sometimes STRING PPI data as "ground truth". They cite the BEELINE paper, but I think it would be extremely important for this MS to clearly state where the ChIP-Seq data comes from, over which samples it was defined (cell-lines? Normal or cancer cell-lines? Sorted bulk cells?). And for instance, why did the authors also not consider regulon databases like e.g. DOROTHEA from Saez-Rodriguez's lab?
3) Definition of EPR is unclear: One clear example of the lack of clarity permeating this MS is in the definition of the EPR metric, and when it is first used in Fig.2. In Methods the authors state that "Early precision is defined as the fraction of true positives in the top k edges (k=the number of edges in the ground truth network by default). Then the early precision rate (EPR) represents the ratio of the early precision value and the early precision for a random predictor for this network. A random predictor's precision is the edge density of the ground-truth network. The EPR measures how well an algorithm is able identify true positive interactions early on in the ranking." So, for instance, in the context of Fig.2 where the authors use non-specific ChIP-Seq data as "ground truth" I am going to assume that k refers to the number of regulatory interactions of a given TF according to ChIP-Seq. If that is the case, then "precision" is surely the wrong term! It should be sensitivity or power, because sensitivity measures the fraction of true positives that are captured. Precision or PPV (positive predictive value) refers to something different, which is the fraction of true positives among all edges that are called "significant/positive" and is related to the FDR via PPV=1-FDR. Likewise, the rate is defined relative to a random predictor, and I can see how this might be related to edge density, but why edge density useful in the context of a TF-regulatory network? It would be in a PPI network but not in a directed bipartite network. For ChIP-Seq data you only have a connectivity right? So, perhaps the authors can make it crystal clear what their definition of EPR is, using the ChIP-Seq data from Fig.2 as an example?
4) Why use non-specific ChIP-Seq data as evaluation framework in Fig.2? Another major concern is why do the authors use non-specific ChIP-Seq data as opposed to specific ChIP-Seq data in a context (differentiation in the hematopoietic system) where specific ChIP-Seq data is readily available?
5) Number of repeats in Fig.2 and elsewhere is insufficient: Another concern I have is whether 10 runs are enough to compare methods? In Fig.2C-D I can see that 10 is enough to establish significance, but some of the differences in Fig.2E-F are not significant or only marginally so, and one wonders how robust these patterns are? Moreover, the number of datapoints in each box of Fig.2E-F is not specified! Same comments apply to all other main figures. Some of the error-bars are missing for other methods, for NetID they are based on potentially an insufficient number of runs, and for other barplots in other figures there are no associated measures of uncertainty e.g. Fig.5C-F.
6) Why was the Granger-causality step not assessed in the context of the simulated data of Fig.3A? I do not see a logical reason why the Granger-causality step could not be evaluated in the context of the simulated data of Fig.3A? Or is it because the simulated data does not explicitly model regulatory interactions? Would it not be worth simulating scRNA-data from toy networks representing regulatory interactions?
7) Scalability analysis is problematic: I don't have a concern regarding the computational efficiency of NetID, but I do have a concern with the lack of clarity in reporting runtimes as shown in Fig.4C. How long a process runs depends on your machine and on the number of cells, both of which are not mentioned in the figure legend. Scalability also requires an analysis of runtime as a function of cell number, and that should be shown in main figure, to see how it scales with cell-number. Third, the authors imply when applying NetID to the mouse kit+ HPC scRNA-Seq data that 40,000 cells is "large". I agree it is large for a method like GENIE3 without a powerful server

etc, but 40,000 is not really that large in the context of current scRNA-Seq datasets. I think that readers would want to know how long NetID would take if run on dataset encompassing ~100,000 cells. This is important for the future practical utility of NetID.
8) Ground truth lineage specific TFs: The authors state that to "asses the performance of NetID in identifying key lineage factors compared to alternative methods, we used previously curated regulators of erythroid and neutrophil fate from the literature as ground truth [38]." Again, this lacks substantial clarity, because how do you justify what is ground-truth here? The cited paper seems to deal with scRNA-Seq data and does not seem to be right reference? Most importantly, are these ground-truth lists of lineage-specific factors derived from single-cell data or sorted bulk-tissue data? Surely, if the authors decide that ChIP-Seq data derived from bulk-samples is an appropriate ground truth, then why not use bulk data of sorted immune-cells (amply available from the Haemosphere/Haemopedia/Immunogenomics projects) as ground truth to define lineage-specific factors? Once again, the lack of clarity in defining your ground-truth is an issue that permeates most of this MS, and is troubling, because the evaluation frameworks used are as important as the methods themselves!
9) Downstream processing of the cell fate matrix F in NetID is not well justified: In F columns represent cell-fates, rows label cells. The authors state that "we first normalized each column of F through division by the sum of the column vector. The purpose of this rescaling step is to compensate for systematically lower cell fate probabilities of rare lineages compared to abundant fates and to make cell fate bias of rare and abundant cell types more comparable. Subsequently, each row is renormalized by dividing by the sum of each row vector." I am deeply concerned about this procedure, because it does not preserve the ranking of cell-fate probabilities for each cell. I can understand the issue of rare cell-fates that have lower probabilities, but surely a few cells will have a higher probability of differentiating into this rare state, otherwise there is something terribly wrong with the Palantir/CellRank method. In my opinion, this issue of rare cell-fates should not even arise in this particular MS, because all biological validations involve fairly well-known cell-fates. So, the rationale or justification for the renormalizing of columns is very unclear. The bottom-line is that the transformation the authors do to the cell-fate matrix does not preserve the ranking of cell-fate probabilities of each cell, and hence this could lead to misleading results. The authors need to resolve this issue.
10) Is regulatory connectivity a justified evaluation metric? Related to the previous point and analysis, I also have a concern as to why the authors think that the regulatory connectivity defined as the sum of the regulatory coefficients is a justified metric to rank TFs? My understanding is that this metric increases with the number of inferred edges, but if a fraction of these edges are false positives, then this would inflate/bias the value for certain TFs. Surely, there must be a more objective way to rank TFs?
11) Is the amount of benchmarking and testing sufficient?: I enjoyed reading the first part of the MS, and I like how the authors went about testing each step of NetID. However, if we want to be rigorous about it, is it really sufficient to compare the impact of say pruning vs non-pruning on just one dataset? My experience tells me that the answer here is no. Often, patterns inferred in one dataset may not generalize in others. So, I think the paper would benefit from a more extensive benchmarking and testing of the methods across more datasets, specially since the paper is being presented as a Methods paper?
12) Seeming arbitrariness in some of the comparisons: in Fig.5F the authors compare to a method called LEAP which however was not included in previous figures e.g. Fig4C or Fig3B-D. There must be a reason for this, but I could not appreciate what it is.
13) Missing literature: Whilst I understand that the purpose of this MS is not to infer regulatory activity, some might argue that it is closely related to the task of inferring a regulatory network. I don't think that the authors need to consider the task of inferring regulatory activity here, because that would go beyond the scope of this MS, but I do think that it would be important in the Introduction and Discussion sections to briefly mention that if the aim is to identify lineage-specific TFs, that an alternative approach is to use regulons to infer regulator activity from which

you can then infer which TFs are more active in one particular lineage. Along these lines, there have been different paradigms, with some recent studies (see Holland C et al Genome Biol 2020, PMID: 32051003, and Wang et al Npj Genomic Medicine 2020, PMID: 32051003) indicating that regulons inferred from integrated resources or bulk-tissue data lead to better inference of TF regulatory activity in scRNA-Seq data compared to approaches like SCENIC that aim to infer regulons from the scRNA-Seq data itself. These studies also concluded that imputation does not help and leads to worse inference. So, I would recommend if the authors were to discuss the above 2 regulon-based approaches in the context of the present MS.

Some minor points:
14) Please define "P" in equation-1 describing the Granger-Causality analysis.
15) Optimization of alpha in eq-3: a concern here is the introduction of this alpha parameter which the authors optimize. But how it is optimized is unclear, and does not seem to enter the discussion in the Results section. Needless to say that tuning alpha to give good results is not justified. Please clearly explain how alpha is optimized and please comment what the actual inferred alpha values are for each application and dataset. Is there much variation? Can't the authors give a guideline as to how to choose alpha?
16) Root cell choice in Palantir: can the authors comment on the robustness of the inference, because selecting as root cell as the one with highest expression of gene "X" could be a suboptimal procedure. At least robustness should be checked right?

Reviewer 2

This paper describes NetID, a method to infer lineage-specific gene regulatory networks from single-cell RNA-sequencing data. NetID features an preprocessing step based on metacells and includes a multi-step process to infer skeleton, lineage-specific and global GRNs. The authors performed benchmarks to evaluate or optimize decisions at several steps with real and simulated datasets. NetID's performance was then demonstrated on real datasets in comparison with existing methods.

This paper has a clear illustration of the major steps of NetID. But the method section does not contain sufficient details to reproduce the results or be evaluated. It is good to see numerous benchmarks included at every stage of the study. However, the actual comparison with existing GRN inference methods is very limited. Lineage-specific gene regulatory network is an exciting direction. It is unclear why NetID ultimately combines them into a final global GRN that loses lineage information and diminishes the gains.

I have the following concerns.

The method section for inferring lineage-specific GRNs is not clear. In line 34 page 15, which metacells are referred to? Please correct typos in Eq 1 and make sure every symbol is defined. What is the values of L and P in Eq 1? Would they cause overfitting? It is unclear how SUMMA performs with only two classifiers. Can the authors show the computed values of alpha in Eq 3? Why is Eq 4 not weighted by the number cells with that fate?

The benchmarking paragraph and Figs 3, 4 give an impression of competing methods, but obviously DCA and others do not provide GRN inference. If the authors compared NetID's metacell generation step against them, it should be clearly stated.

Due to the above confusion, the authors should also clarify whether Figs 5, 6 used existing methods as they are or a part within NetID. Can these standalone methods be included for benchmarking in Figs 3, 4?

Detailed methods for using Dyngen is missing. The authors should clearly describe its parameters and whether the GRN remains unchanged for all cells.

For Explained Expression Variance, could the authors explain why the mean value of R2 over PCs should be used? Each PC contributes a different proportion of total variance. Is this proportion included as a weight for the mean?

Could the authors explain the rationale and methods behind using protein-protein interaction networks for benchmarking GRN inference performance?

The rationale behind obtaining a final global GRN is unclear. Why not simply benchmark and analyze lineage-specific GRNs?

NetID cannot be installed. Error is included below. The authors should carefully test the installation scripts and tutorials in a clean environment.
> install.packages('NetID_0.1.0.tar.gz', repos=NULL, type='source')
ERROR: dependencies 'lmtest', 'RobustRankAggreg', 'irlba', 'rsvd', 'reticulate', 'Seurat', 'Hmisc', 'mclust', 'pracma', 'doParallel', 'doRNG', 'RaceID', 'igraph', 'rARPACK' are not available for package 'NetID'

Authors' response to reviewers

Response to Reviewer #1. The reviewer comments are shown in black and our responses are highlighted in blue. Changes in the revised manuscript are highlighted in red.

Reviewer #1: The paper "Scalable identification of lineage-specific gene regulatory networks from metacells with NetID" by Wang and Gruen presents a novel regulatory network inference algorithm for scRNA-Seq data, called NetID. The algorithm is in effect, an ingenious "stitch-up" of many different methods. One key element is the construction of metacells ,(Baran Y et al Genome Bio 2019) using seeds selected with the "geosketch" algorithm from Berger's lab, although oddly enough this contradicts what is stated in Methods, where the authors imply that a 2nd method implemented in Seurat called "SeuratSketching" is used. The use of meta-cells makes sense as the authors aim to optimize the trade-off between cell-numbers and the sparsity/noise of the profiles used for inference. Once the meta-cells are inferred, the author rely effectively on the published GENIE3 method to infer a global regulatory inference network, which the authors then use as a "scaffold or prior network" to be integrated with lineage-specific networks that are inferred using Granger causality ridge regression. In all honesty though, this Granger- causality step is probably only a minor refinement, because unlike what the authors are trying to imply, there is substantial lineage-specific information in the GENIE3 derived network, as long as GENIE3 is applied sensibly over cell populations where the underlying variation is associated with bifurcating cell-differentiation processes. The authors use both simulated as well as real datasets to assess performance, but the benchmarking is mainly against methods that perform imputation on scRNA-seq data, and not to other existing regulatory network inference methods (see further below). The authors use a number of evaluation frameworks and "ground-truths" but other potentially better alternatives are ignored.

Overall, while I think that NetID should be of interest to the single-cell community, its value seems mostly confined to the construction of metacells and the GENIE3 network, both of which are based on existing algorithms, which would hence seem to justify more of a software or "applications note" paper. Indeed, the "improvement" over existing methods as shown in this MS is only fairly marginal, as there are cases where some of the imputation methods perform better (e.g. Fig4B msHSC-specific ChIP-Seq and non-specific ChIP-Seq), and some benchmarks are

missing. I am also concerned about comparing methods based on only 10 repeated runs. Surely, one should consider more runs to make sure that non- significant differences are truly non-significant. The Granger-causality step method also does not necessarily add much novel biological insight as the TFs that are being recovered are well-known and could have been found by much simpler or other existing methods (e.g. integrating

DEG-analysis or GENIE3 with a PPI-network). Another concern is the lack of clarity in the implementation of methods, as exemplified by the example above when selecting seed cells to build metacells.

Thank you for the constructive feedback. In repsonse to the detailed concerns we improve our benchmarking and better demonstrate the novelty of NetID, in particular, the benefit of using Granger causal modeling for the inference of lineage-specific GRNs.

Below are my major concerns, in the hope these can be useful:

Major concerns:

1) Is the Granger-causality step really needed?: Throughout the MS, the authors seem to imply that the global network inferred with GENIE3 contains no information about lineage- specific regulatory networks, which is why the authors subsequently apply this Granger- causality ridge regression step to seemingly infer lineage-specific regulatory networks. However, I can't possibly agree with this statement. If you apply GENIE3 to a scRNA-Seq data encompassing say a multipotent progenitor population as well as differentiated cells for all downstream lineages, most of the variation in such a dataset would obviously capture

differences between the lineages, and that information gets encoded in the global regulatory network. To then identify lineage specific subnetworks would only be a matter of studying the gene-expression patterns for cells mapping to a given lineage in the context of this global network, which would reveal lineage-specific subnetworks. Hence, the rationale for

Granger-causality ridge regression is not entirely clear to me: I understand that you can use it to infer potential regulatory relations within a lineage, relying on the gradients of expression within a lineage (ordered by pseudotime). However, this would only constitute a refinement. In summary, I feel that the Granger-causality step needs to be benchmarked against an alternative approach which studies the expression patterns of lineage-specific cells on the inferred GENIE3 global network.

Thank you for your valuable suggestions. Firstly, we agree that GENIE3 contains lineage- specific regulatory information. The critical point is how to extract this information from the global network. However, relying solely on gene expression profiles or pseudotime ordering information does not effectively distinguish gene expression across different lineages.

Therefore, it is essential to use CellRank/Palantir cell fate probabilities to provide ordering information and assign cells to lineages.

Moreover, correlating gene expression with cell fate probability can identify more lineage- specific drivers compared to pure differential gene expression analysis [1, 2]. This ordering information helps to disentangle cell lineages. By using each cell fate probability as ordering information for Granger causal correlation analysis, we can achieve lineage-specific gene regulatory network (GRN) inference.

To validate that our method of combining Granger correlation analysis with cell fate probabilities can better infer lineage-specific GRNs, we included the suggested GENIE3 + DEG analysis and the global network inferred by GENIE3 as baselines. Additionally, we evaluated the GRN inference methods SCODE and LEAP, which use pseudotime as ordering information. We benchmarked these methods using the dyngen simulation framework, where the ground truth lineage-specific GRN is known (see updated Method). Our findings indicate that the Granger + cell fate probabilities inference method outperforms all other methods, achieving the highest EPR and AUROC (see updated Fig. 5 and Fig.

S11).

We also benchmarked all five methods on a real human bone marrow dataset. Using Palantir to infer lineage-specific fate probabilities, we inferred a megakaryocyte-specific GRN and used the megakaryocyte-specific ChIP-seq dataset collected by Zhang et al. as the ground truth. Our results show that the Granger + cell fate probabilities method performs better than other methods. Although SCODE had a higher AUROC, its prediction EPR was the lowest among the five methods (see updated Fig. 5 and Fig. S11).

In conclusion, we believe that combining cell fate probabilities with Granger causal correlation analysis can effectively infer lineage-specific GRNs. Alongside the global GRN inference procedure, we believe NetID can provide scalable and accurate network information at both global and lineage-specific levels.

2) Evaluation frameworks lack clarity: the authors sometimes use specific ChIP-seq data, sometimes non-specific ChIP-Seq data and sometimes STRING PPI data as "ground truth". They cite the BEELINE paper, but I think it would be extremely important for this MS to clearly state where the ChIP-Seq data comes from, over which samples it was defined (cell-lines? Normal or cancer cell-lines? Sorted bulk cells?). And for instance, why did the authors also not consider regulon databases like e.g. DOROTHEA from Saez-Rodriguez's lab?

Thanks for your feedback on our evaluation framework. For each dataset in the manuscript, we always use the STRING database and non-specific ChIP-seq datasets as ground truth.

The non-specific ChIP-seq networks were extracted from three resources: DoRothEA, RegNetwork and TTRUST. In DoRothEA, we only considered two levels of evidence: A (curated/high confidence) and B (likely confidence).

For the embryonic and hematopoetic stem cell datasets we also used the mESC and mHSC specific ChIP-seq data provided by BEELINE as ground truth. Each mESC and mHSC specific ChIP-seq data is searched from ENCODE[3], ChIP-Atlas[4] and ESCAPE[5] dataset by the BEELINE pipeline.

We summarize all datasets and ground truth GRN dataset that we used throughout this manuscript in Table S1.

3) Definition of EPR is unclear: One clear example of the lack of clarity permeating this MS is in the definition of the EPR metric, and when it is first used in Fig.2. In Methods the authors state that "Early precision is defined as the fraction of true positives in the top k edges (k=the number of edges in the ground truth network by default). Then the early precision rate (EPR) represents the ratio of the early precision value and the early precision for a random predictor for this network. A random predictor's precision is the edge density of the ground-truth network. The EPR measures how well an algorithm is able identify true positive interactions early on in the ranking." So, for instance, in the context of Fig.2 where the authors use non-specific ChIP-Seq data as "ground truth" I am going to assume that k refers to the number of regulatory interactions of a given TF according to ChIP-Seq. If that is the case, then "precision" is surely the wrong term! It should be sensitivity or power, because sensitivity measures the fraction of true positives that are captured. Precision or PPV (positive predictive value) refers to something different, which is the fraction of true positives among all edges that are called "significant/positive" and is related to the FDR via PPV=1-FDR. Likewise, the rate is defined relative to a random predictor, and I can see how this might be related to edge density, but why edge density useful in the context of a TF-regulatory network? It would be in a PPI network but not in a directed bipartite network. For ChIP-Seq data you only have a connectivity right? So, perhaps the authors can make it crystal clear what their definition of EPR is, using the ChIP- Seq data from Fig.2 as an example?

Early Precision Rate (EPR) is a metric proposed in BEELINE to evaluate early precision compared to a random predictor. EPR is defined as the fraction of true positives among the top-k edges, where k is the number of edges in the ground-truth network. In our benchmark, we consider all predicted top-k edges as positive for all methods, making EPR conceptually similar to precision, which is the fraction of true positive instances among all positive instances.

At the same time, network density is defined as the ratio of the number of predicted edges to the number of all possible edges (calculated as the number of regulators multiplied by the number of targets). Thus, using network density is also applicable to bipartite networks.

Besides, comparing methods using the same ground truth network, the ranking of EPR is not influenced by network density.

We provided a conceptual figure to illustrate the calculation of EPR (revised Figure S1). In this figure, we present a toy example with 2 transcription factors (TFs) and 3 targets.

Ground Truth Network

$$EarlyPrecision = \frac{Hits@K}{K}$$

$$K$$

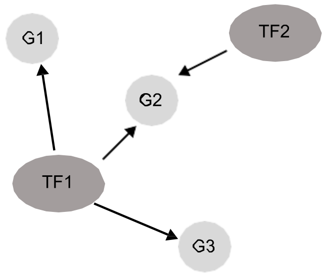

GENIE3 network prediction

Source	Target	True Links	Rank Score
TF1	G1	1	0.9
TF2	G1	0	0.45
TF1	G3	1	0.3
TF2	G2	1	0.1
TF2	G3	0	0.05
TF1	G2	1	0.01

Top K

$$NetworkDensity = \frac{}{MP}$$

$$EPR = \frac{EarlyPrecision}{NetworkDensity}$$

M The number of targets
P the number of regulators
K the number of edges

4) Why use non-specific ChIP-Seq data as evaluation framework in Fig.2? Another major concern is why do the authors use non-specific ChIP-Seq data as opposed to specific ChIP-Seq data in a context (differentiation in the hematopoietic system) where specific ChIP- Seq data is readily available?

In Fig. 2, we use both non-specific ChIP-seq and STRING datasets as ground truth for evaluation. BEELINE also provided a mouse hematopoietic stem cell ChIP-seq dataset. However, the dataset in Fig. 2 from Tusi et al. includes diverse Kit+ cell types such as multipotent progenitors, erythroblasts, and neutrophils, and hematopoietic stem cells are actually rare in this dataset. Hence, the HSC-specific ChiP-seq dataset from BEELINE is unsuitable in this case.

To make our results presented in Fig. 2 more comprehensive and reliable, we added benchmarking for the embryonic stem cell (ESC) and human hematopoiesis datasets, both of which have non-specific ChIP-seq and STRING datasets as ground truth. For ESCs, we compared to an available ESC-specific ChIP-seq-derived ground truth GRN and confirm the observations made with the non-specific ChiP-seq and STRING datasets as ground truth.

The results are presented in revised Figure S2.

5) Number of repeats in Fig.2 and elsewhere is insufficient: Another concern I have is whether 10 runs are enough to compare methods? In Fig.2C-D I can see that 10 is enough to establish significance, but some of the differences in Fig.2E-F are not significant or only marginally so, and one wonders how robust these patterns are? Moreover, the number of datapoints in each box of Fig.2E-F is not specified! Same comments apply to all other main figures. Some of the error-bars are missing for other methods, for NetID they are based on potentially an insufficient number of runs, and for other barplots in other figures there are no associated measures of uncertainty e.g. Fig.5C-F.

Thanks for your observaton. In the updated manuscript, we used 30 repeats for all results in Fig. 2C-D, Fig. 2E-F, Fig. 3B,D , Fig. 4A-B and Fig. 5. For the deterministic gene imputation methods, we

randomly sampled 90% of cells from whole datasets for 30 times to estimate uncertainty. Moreover, the number of datapoints in each box of the figures is specified in the figure legend.

6) Why was the Granger-causality step not assessed in the context of the simulated data of Fig.3A? I do not see a logical reason why the Granger-causality step could not be evaluated in the context of the simulated data of Fig.3A? Or is it because the simulated data does not explicitly model regulatory interactions? Would it not be worth simulating scRNA- data from toy networks representing regulatory interactions?

We agree that the Granger-causal modeling step should also be subject to benchmarking with simulated ground truth data. In the revised Fig. 5A-D, we included a dyngen simulation to benchmark lineage-specific GRN inference. Dyngen explicitly models regulatory interactions, and thus returns a ground truth GRN for each cell. From this we derived a ground truth lineage specific GRN by aggregating regulatory interactions across all cells assigned to a branch associated with a particular terminal state. This benchmarking confirms the superior performance of the Granger-causality inference with the NetID framework compared to alternative mtehods. See update Fig 5 for more details.

7) Scalability analysis is problematic: I don't have a concern regarding the computational efficiency of NetID, but I do have a concern with the lack of clarity in reporting runtimes as shown in Fig.4C. How long a process runs depends on your machine and on the number of cells, both of which are not mentioned in the figure legend. Scalability also requires an analysis of runtime as a function of cell number, and that should be shown in main figure, to see how it scales with cell-number. Third, the authors imply when applying NetID to the mouse kit+ HPC scRNA-Seq data that 40,000 cells is "large". I agree it is large for a method like GENIE3 without a powerful server etc, but 40,000 is not really that large in the context of current scRNA-Seq datasets. I think that readers would want to know how long NetID would take if run on dataset encompassing ~100,000 cells. This is important for the future practical utility of NetID.

We acknowledge that our benchmarking of the computational efficiency required improvement and extended this analysis in the revised manuscript. We aimed to demonstrate that NetID offers significantly better computational efficiency compared to other gene imputation and metacell methods. To better demonstrate the improved computational efficiency of NetID compared to other gene imputation or metacell methods, we used a large embryogenesis atlas dataset for analysis. We selected the E8.25 time point data, which contains approximately 16,000 cells, and performed subsampling with 20%, 40%, 60%, 80%, and 100% of the dataset to evaluate running time. The results show that NetID has excellent scalability and significantly lower running times compared to other methods.

We also evaluated how long NetID would take when applied to a dataset encompassing approximately 105,000 cells. We selected additional time points, including E6.75 to E8.5, and ran NetID for 50 repeats, and compared to the running time when NetID was applied to only ~16,000 cells (E8.25). The results show that NetID's running time increased by only ~7 minutes when applied to ~105,000 cells.

We have updated these results in Fig. 4C and also added the computational specifications to the revised manuscript.

8) Ground truth lineage specific TFs: The authors state that to "asses the performance of NetID in identifying key lineage factors compared to alternative methods, we used previously curated regulators of erythroid and neutrophil fate from the literature as ground truth [38]." Again, this lacks substantial clarity, because how do you justify what is ground- truth here? The cited paper seems to deal with scRNA-Seq data and does not seem to be right reference? Most importantly, are these ground-truth lists of lineage-specific factors derived from single-cell data or sorted bulk-tissue data? Surely, if the authors decide that ChIP-Seq data derived from bulk-samples is an appropriate ground truth, then why not use bulk data of sorted immune-cells (amply available from the Haemosphere/Haemopedia/Immunogenomics projects) as ground truth to define

lineage- specific factors? Once again, the lack of clarity in defining your ground-truth is an issue that permeates most of this MS, and

is troubling, because the evaluation frameworks used are as important as the methods themselves!

We agree that clarity needs to be improved.

The regulatory pathways underpinning hematopoetic differentiation are relatively well studied, and we only refer to those regulators with known functions during erythorid and neutrophil differentiation.

Among the selected erythroid-lineage related transcription factors, *Gata1* plays a pivotal role by activating erythroid-specific genes and repressing non-erythroid lineage genes [6]. *Zfpm1* (also known as *FOG1*) partners with *Gata1* to enhance its function [7]. *Gfi1b* further supports erythroid differentiation by repressing genes that promote alternative lineages and maintaining the quiescence of progenitor cells [8]. *Klf1* (EKLF) is crucial for the expression of β-globin and other erythroid-specific genes [9]. *Ldb1* forms a complex with *Gata1* and *Tal1* (Scl) to regulate the expression of critical erythroid genes [10]. *Tal1* is essential for the establishment of the erythroid lineage by regulating the expression of genes involved in erythroid progenitor proliferation and survival [11].

For the neutrophil lineage, we refer to *Spi1* (PU.1), which is a master regulator of myeloid lineage differentiation [12], promoting the expression of genes necessary for neutrophil development and function. *Irf8* is critical in the early stages of neutrophil differentiation, where it acts alongside *Spi1* to drive the expression of myeloid-specific genes [13]. *Irf5* further supports this process by regulating genes involved in inflammatory responses and neutrophil function [14]. *Zeb2* plays a crucial role in the terminal differentiation of neutrophils by repressing genes that promote alternative cell fates, thereby ensuring commitment to the neutrophil lineage [15]. *Klf4* is involved in the later stages of differentiation, where it enhances the expression of genes crucial for neutrophil maturation and function [16].

We added these ground truth lineage specifc TFs information in Table S2, and updated references in our revised manuscript.

9) Downstream processing of the cell fate matrix in NetID is not well justified: In F columns represent cell-fates, rows label cells. The authors state that "we first normalized each column of through division by the sum of the column vector. The purpose of this rescaling step is to compensate for systematically lower cell fate probabilities of rare lineages compared to abundant fates and to make cell fate bias of rare and abundant cell types more comparable. Subsequently, each row is renormalized by dividing by the sum of each row vector." I am deeply concerned about this procedure, because it does not preserve the ranking of cell-fate probabilities for each cell. I can understand the issue of rare cell-fates that have lower probabilities, but surely a few cells will have a higher probability of differentiating into this rare state, otherwise there is something terribly wrong with the Palantir/CellRank method. In my opinion, this issue of rare cell-fates should not even

arise in this particular MS, because all biological validations involve fairly well-known cell- fates. So, the rationale or justification for the renormalizing of columns is very unclear. The bottom-line is that the transformation the authors do to the cell-fate matrix does not preserve the ranking of cell-fate probabilities of each cell, and hence this could lead to misleading results. The authors need to resolve this issue.

Thank you for raising this valid concern. We have removed this renormalization step and updated all results in the manuscript accordingly.

10) Is regulatory connectivity a justified evaluation metric? Related to the previous point and analysis, I also have a concern as to why the authors think that the regulatory connectivity defined as the sum of the regulatory coefficients is a justified metric to rank TFs? My understanding is that this metric increases with the number of inferred edges, but if

a fraction of these edges are false positives, then this would inflate/bias the value for certain TFs. Surely, there must be a more objective way to rank TFs?

We appreciate the insightful questions raised regarding the justification of regulatory connectivity as an evaluation metric in our study. To address this concern, we have carefully considered the following points:

1. In our approach using the GENIE3 method, we mitigate the impact of false positives in regulatory edges by adopting stringent criteria. Specifically, we limit our analysis to the top 50 targets of each transcription factor (TF) and filter out edges with low importance (< 0.001). This approach aims to prioritize stronger regulatory relationships, thereby minimizing the inclusion of spurious connections that could inflate the regulatory connectivity metric.

2. To further enhance the objectivity of our TF ranking metric, we acknowledge the potential bias introduced by simply summing regulatory coefficients. Instead, we are using the mean coefficient across inferred edges for each TF. This adjustment ensures that the metric is not disproportionately influenced by the number of edges, thereby providing a more balanced representation of regulatory influence with reduced impact of false positive connections.

The new results were added to revised Fig. 6C-E.

11) Is the amount of benchmarking and testing sufficient?: I enjoyed reading the first part of the MS, and I like how the authors went about testing each step of NetID. However, if we want to be rigorous about it, is it really sufficient to compare the impact of say pruning vs non-pruning on just one dataset? My experience tells me that the answer here is no. Often, patterns inferred in one dataset may not generalize in others. So, I think the paper would benefit from a more extensive benchmarking and testing of the methods across more datasets, specially since the paper is being presented as a Methods paper?

Thank you for your positive feedback and thoughtful questions regarding the benchmarking and testing of NetID.

We have incorporated additional datasets including ESC and human hematopoiesis datasets (revised Fig. S4-6) into this part of the benchmarking analysis. This expansion aims to evaluate the robustness and generalizability of NetID across diverse biological contexts.

Each dataset now includes comparisons against at least two distinct ground truth networks. This ensures comprehensive testing and validation of our method against various reference standards, thereby enhancing the reliability of our findings.

12) Seeming arbitrariness in some of the comparisons: in Fig.5F the authors compare to a method called LEAP which however was not included in previous figures e.g. Fig4C or Fig3B-D. There must be a reason for this, but I could not appreciate what it is.

Thank you for your insightful observation regarding the comparisons in Figure 5F and the inclusion of LEAP. We appreciate the opportunity to clarify the rationale behind our methodology:

Each figure in our manuscript serves a specific purpose to compare different aspects of our NetID methodology. Figures 4 and 3 (panels B-D) primarily focus on benchmarking NetID against other imputation methods and metacell approaches. This comparison is crucial to demonstrate the efficacy of our strategy in accurately estimating Gene Expression Profiles (GEP), thereby enhancing the stability of Gene Regulatory Network (GRN) inference.

In Figure 5, our aim shifts towards evaluating lineage-specific GRN inference methods utilizing pseudotime or velocity-derived fate probabilities. The inclusion of methods like SCODE and LEAP specifically addresses their reliance on dynamic (pseudotime) information. This allows us to compare NetID's performance in lineage-specific GRN inference against methodologies that incorporate dynamics in different ways. Hence, the inclusion of LEAP in Figure 5F aligns with this objective to provide a comprehensive evaluation of methods utilizing dynamic information in GRN inference contexts.

We have added this information to the revised manuscript to clearly state the purpose of each benchmark.

13) Missing literature: Whilst I understand that the purpose of this MS is not to infer regulatory activity, some might argue that it is closely related to the task of inferring a regulatory network. I don't think that the authors need to consider the task of inferring regulatory activity here, because that would go beyond the scope of this MS, but I do think that it would be important in the Introduction and Discussion sections to briefly mention that if the aim is to identify lineage-specific TFs, that an alternative approach is to use regulons to infer regulator activity from which you can then infer which TFs are more active in one particular lineage. Along these lines, there have been different paradigms, with some recent studies (see Holland C et al Genome Biol 2020, PMID: 32051003, and Wang et al Npj Genomic Medicine 2020, PMID: 32051003) indicating that regulons inferred from integrated resources or bulk-tissue data lead to better inference of TF regulatory activity

in scRNA-Seq data compared to approaches like SCENIC that aim to infer regulons from the scRNA-Seq data itself. These studies also concluded that imputation does not help and leads to worse inference. So, I would recommend if the authors were to discuss the above 2 regulon-based approaches in the context of the present MS.

We followed your suggestion by adding the following statement to the revised discussion:

“We note that future extensions of NetID could draw from previous approaches to overcome the issue of sparsity of scRNA-seq data for identifying lineage-specific regulators. In particular, incorporation of regulon activity could help to prioiritize key transcription factors driving lineage-specific GRN modules [51,52]“

Some minor points:

14) Please define "P" in equation-1 describing the Granger-Causality analysis.

As stated in the methods section, P denotes the number of regulators of target gene i.

15) Optimization of alpha in eq-3: a concern here is the introduction of this alpha parameter which the authors optimize. But how it is optimized is unclear, and does not seem to enter the discussion in the Results section. Needless to say that tuning alpha to give good results is not justified. Please clearly explain how alpha is optimized and please comment what the actual inferred alpha values are for each application and dataset. Is there much variation? Can't the authors give a guideline as to how to choose alpha?

Thank you for your raising this concern. We originally implemented this parameter aimed to combine GENIE3 coefficients and Granger coefficients. However, since we already use the GENIE3 skeleton, a combination of coefficients a posteriori may not even be necessary.

Therefore, we decided to simplify NetID and directly regard the GRN inferred from the Granger causal model as the lineage-specific GRN. We revised the Methods section accordingly.

16) Root cell choice in Palantir: can the authors comment on the robustness of the inference, because selecting as root cell as the one with highest expression of gene "X" could be a suboptimal procedure. At least robustness should be checked right?

In Palantir/CellRank, besides root cell selection, we can also manually set the terminal states to make the fate probability inference more stable, leading to more robust GRN inference. To verify this, we conducted two experiments:

1. We randomly sampled one cell from multipotent progenitor cells and calculated lineage-specific GRNs. We repeated this procedure 30 times and calculated the correlation of the inferred GRN coefficients with each other. The results showed that both lineages had overall very stable GRN inference when we specified the terminal states.

2. We selected root cells based on the highest expression of three stem/progenitor marker genes, i.e., *Runx2*, *Cd34*, and *Flit3*. As assessed by GRN correlation, root cell selection based on any of these genes provided consistent GRNs, indicating the robustness of GRN inference.

Please see updated Fig. S10 for more details.

Response to Reviewer #2. The reviewer comments are shown in black and our responses are highlighted in blue. Changes in the revised manuscript are highlighted in red.

Reviewer #2: This paper describes NetID, a method to infer lineage-specific gene regulatory networks from single-cell RNA-sequencing data. NetID features an preprocessing step based on metacells and includes a multi-step process to infer skeleton, lineage-specific and global GRNs. The authors performed benchmarks to evaluate or optimize decisions at several steps with real and simulated datasets. NetID's performance was then demonstrated on real datasets in comparison with existing methods.

This paper has a clear illustration of the major steps of NetID. But the method section does not contain sufficient details to reproduce the results or be evaluated. It is good to see numerous benchmarks included at every stage of the study. However, the actual comparison with existing GRN inference methods is very limited. Lineage-specific gene regulatory network is an exciting direction. It is unclear why NetID ultimately combines them into a final global GRN that loses lineage information and diminishes the gains.

Thank you for the constructive feedback. I have the following concerns.

The method section for inferring lineage-specific GRNs is not clear. In line 34 page 15, which metacells are referred to? Please correct typos in Eq 1 and make sure every symbol is defined. What is the values of L and P in Eq 1? Would they cause overfitting? It is unclear how SUMMA performs with only two classifiers. Can the authors show the computed values of alpha in Eq 3? Why is Eq 4 not weighted by the number cells with that fate?

Thanks for pointing out the lack of information in the Methods section, which we addressed in the revised manuscript.

Which metacells are referred to?

We refer to the metacells used to construct the global GRN. This has been clearly stated in the updated Methods section.

Please correct typos in Eq 1 and make sure every symbol is defined

We have corrected the typo in Eq 1 and added the missing definitions.

What is the value of L and P in Eq 1? Would they cause overfitting?

In the Granger causal model, the number of coefficients that need to be estimated for each gene is $P \times L$. P denotes the number of regulators and L denotes the maximum lagged time steps. To avoid overfitting, we applied L2 regularization to the Granger coefficients for each gene. We demonstrate this strategy by focusing on KLF1 and the erythroid lineage as example target gene and lineage, respectively. After sorting the cells by cell fate probabilities, we splited data into training data (80%) and test data (20%). We trained the Granger causal model on training data and evaluated the prediction mean-squared error (MSE) and spearman correlation on test data. Without regularization (λ = 0), the MSE becomes larger and the spearman correlation decreases with increasing P and L. As the regularization strength λ is increased, the MSE is reduced and

spearman correlation improves (revised Fig. S11A-B). Based on our trials we set λ = 150 as the default.

It is unclear how SUMMA performs with only two classifiers. Can the authors show the computed values of alpha in Eq 3?

We have carefully considered both reviewers' concerns. We decided to remove the linear combination to marginally refine the global network and use the Granger-causal modeling

only for lineage-specific GRN inference in the revised manuscript. We updated the Methods section accordingly.

Why is Eq 4 not weighted by the number of cells with that fate?

In Eq 4, we aimed to refine the final global network by adding cell fate information. However, there is no clear mathematical implication that the optimal weight is exactly the same as or linearly correlated with the number of cells of that fate. In the original manuscript, we considered each lineage to be equally important for the global GRN. We do agree that it may not be beneficial to merge those lineage-specific GRNs again to obtain a refined global GRN since the choice of the weights is unclear. We therefore use the Granger-causal modeling only for lineage-specific GRN inference in the revised manuscript. We updated the Methods section accordingly.

The benchmarking paragraph and Figs 3, 4 give an impression of competing methods, but obviously DCA and others do not provide GRN inference. If the authors compared NetID's metacell generation step against them, it should be clearly stated.

We apologize for the lack of clarity in describing our benchmarking strategy. In Fig. 3 and 4, we compared NetID's metacell generation step against other imputation method, and we clearly state this at the beginning of the section in the revised manuscript.

Due to the above confusion, the authors should also clarify whether Figs 5, 6 used existing methods as they are or a part within NetID. Can these standalone methods be included for benchmarking in Figs 3, 4?

In the revised Fig. 5, we aim to benchmark the Granger-Causal part of NetID against other methods for lineage-specific GRN inference. Each method used metacell gene expression profiles generated by NetID to infer lineage-specific GRNs. However, in Figs. 3 and 4, we primarily benchmark global GRNs and compare NetID with other imputation methods to validate that use of metacells is beneficial. Therefore, Figs. 3, 4, and 5 examine the methods from different perspectives. We clearly state the aim of the benchmarking presented in Fig. 5 at the beginning of the section.

Detailed methods for using Dyngen is missing. The authors should clearly describe its parameters and whether the GRN remains unchanged for all cells.

For the Dyngen simulation we have updated the descriptiopn of simulation parameters in the revised method section. The Dyngen simulation generates a global GRN skeleton. At the same time it also returns cell-specific GRNs [17].

Since we use GENIE3 to infer a global GRN in Fig. 4, we focus on the global GRN skeleton returned by the dyngen simulation as the ground truth. In Fig. 5, we used cell-specific GRNs returned by dyngen, and aggregate the cell specific GRNs for each lineage to define a ground truth for the lineage-specific GRN.

All above details are included in the updated methods and the code to conduct the the dyngen simulation for this manuscript is accessible in our github repository (https://github.com/WWXkenmo/NetID_package/blob/main/dyngen_simulation_netID.r)

For Explained Expression Variance, could the authors explain why the mean value of R2 over PCs should be used? Each PC contributes a different proportion of total variance. Is this proportion included as a weight for the mean?

In response to this concern we reconsidered our strategy. In the revised version, we have updated the results and instead of using the mean value of R2 over PCs we now use the proportion of total variance as weights to obtain a weighted explained expression variance.

Could the authors explain the rationale and methods behind using protein-protein interaction networks for benchmarking GRN inference performance?

The STRING network not just contains protein-protein interactions, but various sources indicating functional interactions or associations. Many GRN methods including GENIE3 do not just predict direct interactions, but also indirect ones [18]. Therefore, comparison to STRING informs on the frequency of interactions with additional evidence for direct interactions.

The rationale behind obtaining a final global GRN is unclear. Why not simply benchmark and analyze lineage-specific GRNs?

We agree that the integration of the lineage-specific GRNs to a refined global GRN is not well justified and it is unclear how to determine the weights for combining lineage-specific GRNs. Therefore, we followed your recommendation and removed this step.

Instead, we added benchmarking of lineage-specific GRNs on both simulated and real ground truth datasets in Fig.5

NetID cannot be installed. Error is included below. The authors should carefully test the installation scripts and tutorials in a clean environment.

> install.packages('NetID_0.1.0.tar.gz', repos=NULL, type='source')

ERROR: dependencies 'lmtest', 'RobustRankAggreg', 'irlba', 'rsvd', 'reticulate', 'Seurat', 'Hmisc', 'mclust', 'pracma', 'doParallel', 'doRNG', 'RaceID', 'igraph', 'rARPACK' are not available for package 'NetID'

Please follow the installation tutorial on our github page to ensure you successful installation of the package. We have tested the installation in a fresh and clean conda environment.

In the current version, dependencies need to be installed manually. We are currently preparing a package for submission to CRAN to increase accessibility.

References

1. Weiler P, Lange M, Klein M, Pe'er D, Theis F: **CellRank 2: unified fate mapping in multiview single-cell data.** *Nat Methods* 2024.
2. Lange M, Bergen V, Klein M, Setty M, Reuter B, Bakhti M, Lickert H, Ansari M, Schniering J, Schiller HB: **CellRank for directed single-cell fate mapping.** *Nature methods* 2022, **19:**159-170.
3. de Souza N: **The ENCODE project.** *Nat Methods* 2012, **9:**1046.
4. Oki S, Ohta T, Shioi G, Hatanaka H, Ogasawara O, Okuda Y, Kawaji H, Nakaki R, Sese J, Meno C: **ChIP-Atlas: a data-mining suite powered by full integration of public ChIP-seq data.** *EMBO Rep* 2018, **19**.

5. Xu H, Baroukh C, Dannenfelser R, Chen EY, Tan CM, Kou Y, Kim YE, Lemischka IR, Ma'ayan A: **ESCAPE: database for integrating high-content published data collected from human and mouse embryonic stem cells.** *Database (Oxford)* 2013, **2013:**bat045.

6. Ferreira R, Ohneda K, Yamamoto M, Philipsen S: **GATA1 function, a paradigm for transcription factors in hematopoiesis.** *Mol Cell Biol* 2005, **25:**1215-1227.

7. Mancini E, Sanjuan-Pla A, Luciani L, Moore S, Grover A, Zay A, Rasmussen KD, Luc S, Bilbao D, O'Carroll D, et al: **FOG-1 and GATA-1 act sequentially to specify definitive megakaryocytic and erythroid progenitors.** *EMBO J* 2012, **31:**351-365.

8. Vassen L, Beauchemin H, Lemsaddek W, Krongold J, Trudel M, Moroy T: **Growth factor independence 1b (gfi1b) is important for the maturation of erythroid cells and the regulation of embryonic globin expression.** *PLoS One* 2014, **9:**e96636.

9. Tallack MR, Whitington T, Yuen WS, Wainwright EN, Keys JR, Gardiner BB, Nourbakhsh E, Cloonan N, Grimmond SM, Bailey TL, Perkins AC: **A global role for KLF1 in erythropoiesis revealed by ChIP-seq in primary erythroid cells.** *Genome Res* 2010, **20:**1052-1063.

10. Love PE, Warzecha C, Li L: **Ldb1 complexes: the new master regulators of erythroid gene transcription.** *Trends Genet* 2014, **30:**1-9.

11. Kassouf MT, Hughes JR, Taylor S, McGowan SJ, Soneji S, Green AL, Vyas P, Porcher C: **Genome-wide identification of TAL1's functional targets: insights into its mechanisms of action in primary erythroid cells.** *Genome Res* 2010, **20:**1064-1083.

12. Smith LT, Hohaus S, Gonzalez DA, Dziennis SE, Tenen DG: **PU. 1 (Spi-1) and C/EBP alpha regulate the granulocyte colony-stimulating factor receptor promoter in myeloid cells.** 1996.

13. Kurotaki D, Yamamoto M, Nishiyama A, Uno K, Ban T, Ichino M, Sasaki H, Matsunaga S, Yoshinari M, Ryo A, et al: **IRF8 inhibits C/EBPalpha activity to restrain mononuclear phagocyte progenitors from di\erentiating into neutrophils.** *Nat Commun* 2014, **5:**4978.

14. Khoyratty TE, Udalova IA: **Diverse mechanisms of IRF5 action in inflammatory responses.** *Int J Biochem Cell Biol* 2018, **99:**38-42.

15. Scott CL, Omilusik KD: **ZEBs: Novel Players in Immune Cell Development and Function.** *Trends Immunol* 2019, **40:**431-446.

16. Shen Y, Hong H, Sangwung P, Lapping S, Nayak L, Zhang L, Jain MK, Liao X: **Kruppel-like factor 4 regulates neutrophil activation.** *Blood Adv* 2017, **1:**662- 668.

17. Cannoodt R, Saelens W, Deconinck L, Saeys Y: **Spearheading future omics analyses using dyngen, a multi-modal simulator of single cells.** *Nature Communications* 2021, **12:**3942.

18. Pratapa A, Jalihal AP, Law JN, Bharadwaj A, Murali T: **Benchmarking algorithms for gene regulatory network inference from single-cell transcriptomic data.** *Nature methods* 2020, **17:**147-154.

Second round of review

Reviewer 1

I think the revised version of this paper is definitely a substantial improvement, and I thank the authors for comprehensively addressing the points raised. I only have two lingering concerns. First, in response to my criticism the authors now acknowledge that the original downstream processing of the cell-fate matrix in NetID was flawed, or at least they have now removed this rescaling step which did not preserve the ranking of cell-fate probabilities. However, the author's response is surprisingly brief, just stating that results were updated, but no comment at all as to the impact of this correction on the actual results. Could the authors please comment in relation to the issue of rare cell-types?
Second, the authors seem to have misunderstood my suggestion to use regulons within this framework, as SCENIC is precisely NOT the method I was recommending, precisely because of the underlying sparsity...In my experience SCENIC "overfits" much more into the random variation of the data and will undoubtedly have more false positives. So in fact, I was suggesting quite the opposite, namely to use regulons that have been inferred using orthogonal datasets (e.g. sorted bulk cells or ChIP-Seq databases) that are therefore not influenced by the sparsity and noise of scRNA-Seq data. I asked the authors to cite two papers, one by Holland C et al Genome Biol 2020, PMID: 32051003, and another by Wang et al Npj Genomic Medicine 2020, PMID: 33083012, but the authors cited the SCENIC paper instead of Wang et al PMID: 33083012. Please remove the SCENIC citation and cite the one by Wang et al to make the point clear!

Reviewer 2

The authors have addressed the majority of my concerns. I recommend the publication of this manuscript provided the authors have addressed the concerns from the other reviewer and my minor concerns below:

Can the authors report the exact values of L and P used in this study?

More method details are needed for simulation with Dyngen as it supports major conclusions of this paper. The method section is supposed to include sufficient details without the need to look up from external sources. Were there any nondefault parameters used? How were cell-specific GRNs aggregated into lineage-specific GRNs?

Updates of Explained Expression Variance are not reflected in Methods.

Authors' response to reviewers

Response to Reviewer #1. The reviewer comments are shown in black and our responses are highlighted in blue. Changes in the revised manuscript are highlighted in red.

Reviewer #1: I think the revised version of this paper is definitely a substantial improvement, and I thank the authors for comprehensively addressing the points raised. I only have two lingering concerns.
First, in response to my criticism the authors now acknowledge that the original downstream processing of the cell-fate matrix in NetID was flawed, or at least they have now removed this rescaling step which did not preserve the ranking of cell-fate probabilities. However, the author's response is surprisingly brief, just stating that results were updated, but no comment

at all as to the impact of this correction on the actual results. Could the authors please comment in relation to the issue of rare cell-types?

The primary purpose of using the cell-fate matrix is to provide cell ranking information when we apply the Granger causal regression model. However, rescaling the rank matrix column and performing row renormalization may scramble the cell fate probability matrix as noted by this reviewer. The adjustment of the preprocessing steps leads to the difference of inferred GRN weights.

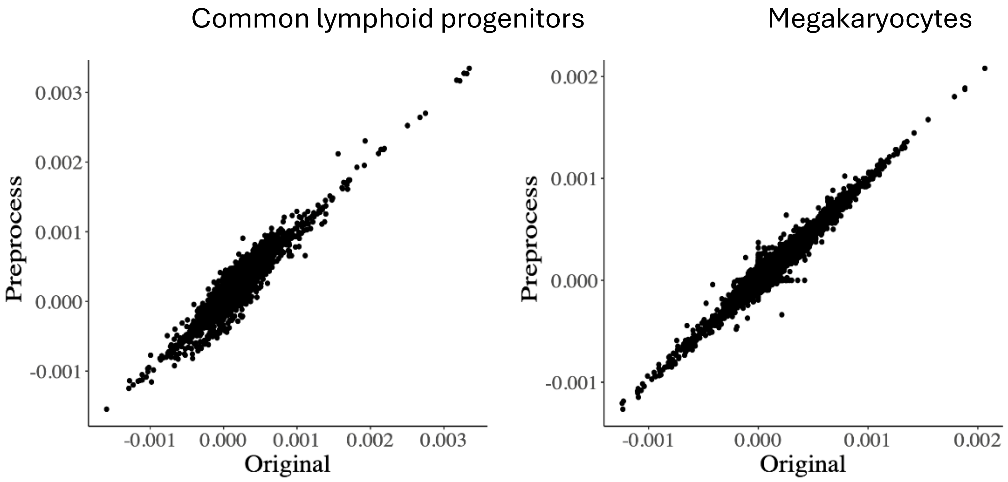

Here, we performed GRN inference using the human hematopoiesis dataset (used in Fig. 5). We used the original (X-axis) and preprocessed (Y-axis) cell fate probability matrix to perform GRN weights inference. Although Megakarocytes are the rarest cell type in this dataset (<1%), we observed that GRN weights exhibit only limited dependence on the preprocessing procedure (right), similar to the behaviour observed for common lymphoid progenitors (left).

Moreover, we tested whether the original preprocessing steps better identified rare lineage associate genes. Following the cell fate inference in Fig. 5E, we applied preprocessing on the cell fate probability matrix of the hematopoiesis datasets and tested if this procedure could better identify the lineage-specific markers. We curated the ground truth gene list from the CellMarker 2.0 database. We ranked the genes by calculating the correlation between gene expression and Megakrocyte cell fate probability. We observed that the preprocessing indeed leads to a deterioration in the identification of rare lineage associate signatures.

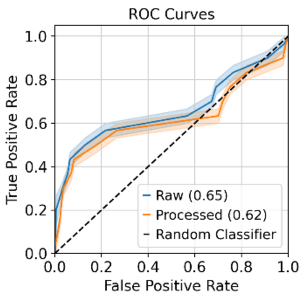

Without the rescaling procedure, NetID still gives excellent performance when inferring lineage-specific GRNs of a rare lineage (see Fig. 5E). Therefore, we followed the recommendation of the

reviewer and use the raw cell fate probability matrix inferred by Palantir/Cellrank to perform downstream analysis.

Second, the authors seem to have misunderstood my suggestion to use regulons within this framework, as SCENIC is precisely NOT the method I was recommending, precisely because of the underlying sparsity…In my experience SCENIC "overfits" much more into the random variation of the data and will undoubtedly have more false positives. So in fact, I was suggesting quite the opposite, namely to use regulons that have been inferred using orthogonal datasets (e.g. sorted bulk cells or ChIP-Seq databases) that are therefore not influenced by the sparsity and noise of scRNA-Seq data. I asked the authors to cite two papers, one by Holland C et al Genome Biol 2020, PMID: 32051003, and another by Wang et al Npj Genomic Medicine 2020, PMID: 33083012, but the authors cited the SCENIC paper instead of Wang et al PMID: 33083012. Please remove the SCENIC citation and cite the one by Wang et al to make the point clear!

We apologize for this misunderstanding. From your previous comment we failed to find the work of Wang et al. because the same PMID (32051003) was given for both references. Hence, we focused on intepreting regulon activity rather than stressing the importance of incorporating different modality information to improve regulon activity inference. We have updated the references and amended our discussion as following contents:

"In particular, incorporation of orthogonal datasets that are less affected by sparsity, such as bulk RNA- and ATAC-seq as well as ChiP-seq data, to infer regulon activity could help to prioritize key transcription factors driving lineage-specific GRN modules [52, 53]".

Response to Reviewer #2. The reviewer comments are shown in black and our responses are highlighted in blue. Changes in the revised manuscript are highlighted in red.

Reviewer #2: The authors have addressed the majority of my concerns. I recommend the publication of this manuscript provided the authors have addressed the concerns from the other reviewer and my minor concerns below:

Can the authors report the exact values of L and P used in this study?

Our computational experiments (Fig. S11) indicates that using a large regularization parameter P and a sufficiently large parameter L leads to a consistently better performance. Therefore, in this study, we fixed L = 30 and P = 150 for all analyses. We added this information in the Results and Methods section.

More method details are needed for simulation with Dyngen as it supports major conclusions of this paper. The method section is supposed to include sufficient details without the need to look up from external sources. Were there any nondefault parameters used? How were cell-specific GRNs aggregated into lineage-specific GRNs?

We performed the dyngen simulation entirely using default parameters and we now clearly state this point in our updated methods section along with more details describing the simulation:

"Each dataset simulates 50 TFs, 200 targets and 50 housekeeping genes, in 4,000 cells. For all other parameters we used the default setting of dyngen.

In Fig. 5, we utilized dyngen to simulate bifurcating topology scRNA-seq data with cell-specific ground truth GRNs. To define lineage-specific GRNs, we aggregated all cell-specific GRNs for lineages CT1 or CT2, respectively. To obtain the aggregated GRN for a cell type, e.g. CT1, we sum up the cell-specific network of all cells belonging to this lineage:

$$GRN_{CT1} = \sum_{i \in S(CT1)} CSN_i \,, \tag{8}$$

$S(CT1)$ denotes the set of all cells belonging to the CT1 terminal states, and CSN_i denotes the cell-specific network of cell *i*."

Updates of Explained Expression Variance are not reflected in Methods.

Thanks for your kind reminder. We have updated the detailed explanation of Explained Expression Variance as follows:

“For each PC, we evaluate a regression model with the sampled cells as predictors, and calculate the goodness-of-fit (R2). The EEV value is define as follow:

$$EEV = \sum_{i=1}^{10} \frac{\lambda_i}{\sum_{i=1}^{10} \lambda_i} R_i^2 \,, \tag{7}$$

Where λ_i is the eigenvalue of i-th PC.”

Third round of review

Reviewer 1

I am happy with revisions made
